# Concept and application of a computational vaccinology workflow

**DOI:** 10.1186/1745-7580-6-S2-S7

**Published:** 2010-11-03

**Authors:** Johannes Söllner, Andreas Heinzel, Georg Summer, Raul Fechete, Laszlo Stipkovits, Susan Szathmary, Bernd Mayer

**Affiliations:** 1 emergentec biodevelopment GmbH, Rathausstrasse 5/3, 1010 Vienna, Austria; 2 University of Applied Sciences, Softwarepark 11, 4232 Hagenberg, Austria; 3 RT-Europe Research Center, 9200 Mosonmagyarovar, Var 2, Hungary; 4 Galenbio Kft., Erdőszél köz 21, 1037 Budapest, Hungary and GalenBio, Inc., 5922 Farnsworth Ct, Carlsbad, CA 92008, USA; 5 Institute for Theoretical Chemistry, University of Vienna, Währinger Strasse 17, 1090 Vienna, Austria

## Abstract

**Background:**

The last years have seen a renaissance of the vaccine area, driven by clinical needs in infectious diseases but also chronic diseases such as cancer and autoimmune disorders. Equally important are technological improvements involving nano-scale delivery platforms as well as third generation adjuvants. In parallel immunoinformatics routines have reached essential maturity for supporting central aspects in vaccinology going beyond prediction of antigenic determinants. On this basis computational vaccinology has emerged as a discipline aimed at *ab-initio* rational vaccine design.

Here we present a computational workflow for implementing computational vaccinology covering aspects from vaccine target identification to functional characterization and epitope selection supported by a Systems Biology assessment of central aspects in host-pathogen interaction. We exemplify the procedures for Epstein Barr Virus (EBV), a clinically relevant pathogen causing chronic infection and suspected of triggering malignancies and autoimmune disorders.

**Results:**

We introduce pBone/pView as a computational workflow supporting design and execution of immunoinformatics workflow modules, additionally involving aspects of results visualization, knowledge sharing and re-use. Specific elements of the workflow involve identification of vaccine targets in the realm of a Systems Biology assessment of host-pathogen interaction for identifying functionally relevant targets, as well as various methodologies for delineating B- and T-cell epitopes with particular emphasis on broad coverage of viral isolates as well as MHC alleles.

Applying the workflow on EBV specifically proposes sequences from the viral proteins LMP2, EBNA2 and BALF4 as vaccine targets holding specific B- and T-cell epitopes promising broad strain and allele coverage.

**Conclusion:**

Based on advancements in the experimental assessment of genomes, transcriptomes and proteomes for both, pathogen and (human) host, the fundaments for rational design of vaccines have been laid out. In parallel, immunoinformatics modules have been designed and successfully applied for supporting specific aspects in vaccine design. Joining these advancements, further complemented by novel vaccine formulation and delivery aspects, have paved the way for implementing computational vaccinology for rational vaccine design tackling presently unmet vaccine challenges.

## Background

Immunological applications of computational biology date back to the roots of the field, e.g. for deriving hydrophilicity profiles based on the primary protein sequence and relating these profiles to B-cell antigenicity [1]. While modern immunoinformatics is not as burgeoning as other areas of bioinformatics (in particular to note the omics field) there is a well established community for traversing models of immune responses into the world of translational research and application. Recent success examples of immunoinformatics include contributions to the understanding of H1N1 immunity [2] and methods to predict determinants of cellular immune responses for potentially every sequenced class I HLA-A and -B variant [3]. Even more recently, reviews aimed at highlighting important concepts of the emerging area of computational vaccinology have been presented [4]. While definitions of computational vaccinology vary, a consensus may be formulated as ‘computational technologies dedicated to supporting and improving development of vaccines’. 

This work intends to point out classical as well as novel components relevant to computational vaccinology. We have chosen an example workflow as the vehicle to convey a basic scaffold and practical application examples for rational vaccine design. While there has been some argument concerning the principal feasibility of rational vaccine design [5] we demonstrate procedures on how computational methods can be harnessed to streamline the process of vaccine R&D, to reduce development cost and time, and to ultimately increase probability of success in formulating novel as well as improve existing vaccines. 

Computational vaccinology embodies a complex collection of (bio)informatics, where a number of core areas can be identified [4]. One of these involves methods necessary for understanding the function of proteins and genes including, as invigorated by next generation sequencing technologies, assembly and annotation of genomes. These methods have recently been complemented by computational Systems Biology approaches with the aim to infuse static biological objects with the notion of context not only for providing a better understanding of a pathogen but specifically for analyzing host-pathogen interaction. A second major element, following a reductionist approach, confers to epitope (immune determinant) prediction for delineating targets of immune responses at highest possible resolution [6].

Due to the inherent complexity of this field with respect to methodologies applied and for integrating existing as well as generated data in tight connection with experimentalists methods for knowledge management and remote collaboration become inevitable. While not strictly bioinformatical in nature these components resemble important aspects of computational vaccinology via fostering an integration of results of involved bioinformatics and wet lab work into the larger context of integrated, highly multidisciplinary research and development. In this second field numerous generic components such as WIKIs or other collaborative solutions can be used. In this context we in particular propose network based data viewers which, although inherently generic, appear particularly well suited to support heterogeneous data landscapes as found in vaccinology in general. 

We use Epstein Barr Virus (EBV) as a model for exemplifying elements of a computational vaccinology workflow, as this pathogen shows substantial clinical relevance. However, no broadly applicable vaccine has been developed so far [7,8].

As a DNA virus causing chronic infection potentially associated with numerous neoplasms and autoimmune disorders EBV belongs to a new class of hard to manage pathogens. This group is characterized by a frequently chronic, immune-modulatory or immuno-evasive phenotype imposing particular pitfalls when applying traditional vaccine approaches. On the other hand these pathogens may become addressable by utilizing emerging vaccine technologies [9-11], eventually in combination with alternative approaches such as peptide vaccines [12-15].

Next to providing an overview of major building blocks valuable in computational vaccinology we focus in the following on the concept of workflows. Although generic in nature we want to stress the particular importance of computational workflows for integration of heterogeneous methods and data as found in computational vaccinology. Here numerous and highly specialized immunoinformatics as well as more general bioinformatics tools come into play, which in their totality require temporal and spatial organization. Temporal organization refers to the need for following a structured process, e.g. when output of one functional module is default input for the subsequent method. Spatial organization refers to the need for distributed computing for efficiently handling tasks. We discuss our workflow design approach utilizing the Taverna platform [16]. While workflows are important for structuring R&D processes their interim results data can be utterly overwhelming just by the sheer amount and heterogeneity of output generated. We therefore address specific issues to be considered when implementing a bioinformatics workflow for control, organization and data/results visualization supporting computational vaccinology.

## Results

### Workflow overview

**Figure 1:**
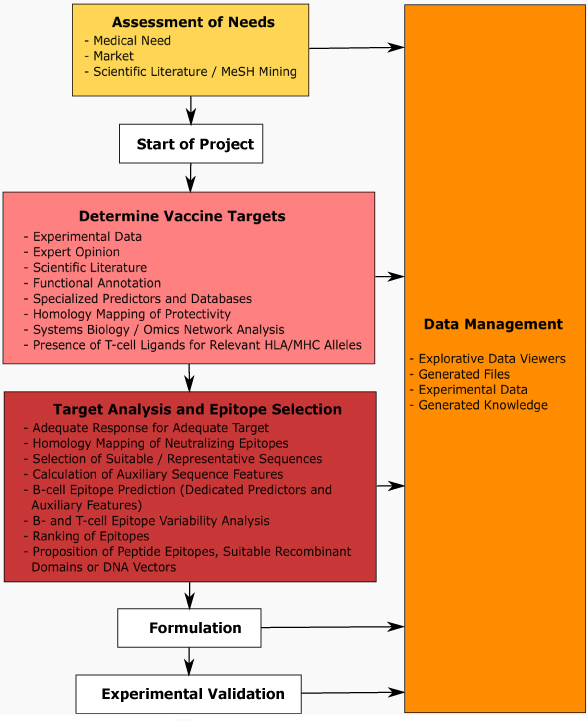
**Prototypic representation of a (computational) vaccine design workflow.** The scheme spans the entire pre-clinical project life cycle from concept phase, determination of vaccine targets, further to detailed epitope analysis, formulation, and experimental validation. The entire process is optimally embedded in an integrated data and knowledge management framework.

We in the following present an example workflow for a vaccine design R&D project centrally resting on computational vaccinology. We define scope, functional modules and a set of technologies relevant in this context, as schematically presented in Figure 1. 

Evident from Figure 1, computational vaccinology resembles comparable procedure as applied in any vaccine design project. Before project start the medical, scientific and application perspective needs to be analyzed. Informatics provides a number of tools already supporting this early phase. Generic knowledge management systems, in particular WIKIs, facilitate handling of literature and results sharing in particular in a distributed, collaborative research environment. Next to supporting knowledge management, tools from literature mining are well suited for identifying and analyzing routes to take regarding the design of a vaccine for a specific pathogen. For example, NCBI MeSH terms (http://www.nlm.nih.gov/pubs/factsheets/mesh.html), a controlled, hierarchical biomedical vocabulary associated with all publications in Medline, allow retrieval of pathogen-to-disease associations. In the case of EBV e.g. associations with autoimmune diseases as well as cancer are revealed by identification of co-occurrence of pathogen and disease categories as found in the scientific literature. Indeed, EBV has been associated with numerous autoimmune disorders and malignancies as summarized in Table 1. 

**Table 1:** Association between autoimmune diseases, neoplasms and pathogens as found by NCBI MeSH.

pathogen	autoimmune disease	# co-occurrence	pathogen	neoplasm	# co-occurrence
Measles virus	Multiple Sclerosis	384	Papillomaviridae	Uterine Cervical Neoplasms	5854
**Herpesvirus 4, Human**	Arthritis, Rheumatoid	279	Helicobacter pylori	Stomach Neoplasms	3231
Campylobacter jejuni	Guillain-Barre Syndrome	167	Papillomaviridae	Carcinoma, Squamous Cell	2252
Enterovirus B, Human	Diabetes Mellitus, Type 1	164	Papillomaviridae	Cervical Intraepithelial Neoplasia	1922
Campylobacter jejuni	Polyradiculoneuropathy	150	**Herpesvirus 4, Human**	Cell Transformation, Viral	1899
**Herpesvirus 4, Human**	Multiple Sclerosis	136	**Herpesvirus 4, Human**	Burkitt Lymphoma	1853
**Herpesvirus 4, Human**	Lupus Erythematosus, Systemic	126	Hepatitis B virus	Liver Neoplasms	1673
Helicobacter pylori	Purpura, Thrombocytopenic, Idiopathic	124	Hepatitis B virus	Carcinoma, Hepatocellular	1615
Theilovirus	Multiple Sclerosis	118	**Herpesvirus 4, Human**	Nasopharyngeal Neoplasms	1518
Herpesvirus 6, Human	Multiple Sclerosis	113	Herpesvirus 8, Human	Sarcoma, Kaposi	1391
Mycobacterium tuberculosis	Arthritis, Rheumatoid	110	Hepacivirus	Carcinoma, Hepatocellular	1005
Escherichia coli	Arthritis, Rheumatoid	101	Hepacivirus	Liver Neoplasms	996
Chlamydophila pneumoniae	Multiple Sclerosis	74	**Herpesvirus 4, Human**	Hodgkin Disease	975
Streptococcus pyogenes	Arthritis, Rheumatoid	61	Simian virus 40	Cell Transformation, Viral	860
Human T-lymphotropic virus 1	Multiple Sclerosis	61	**Herpesvirus 4, Human**	Lymphoma	753
Lymphocytic choriomeningitis virus	Diabetes Mellitus, Type 1	61	Oncogenic Viruses	Neoplasms	720
Rubella virus	Multiple Sclerosis	61	Helicobacter pylori	Lymphoma, B-Cell, Marginal Zone	704

Table 1 lists the most frequent co-occurrences found between disease (autoimmune disorders and malignancies) and pathogen terms according to MeSH term mining of scientific literature given in Medline. HHV4 (EBV) entries are given in bold.

Certainly additional evidence is needed for verifying such associations, to be retrieved either by applying more subtle text mining methods or ideally by involving domain experts. However, in the case of EBV the literature derived association is supported by various further evidence regarding the etiological link between the virus and prevalent diseases including Multiple Sclerosis and Rheumatoid Arthritis as well as nasopharyngeal neoplasms and lymphomas, although considerable criticism remains regarding these associations [17,18].

After clarifying generic project boundaries the further procedures can be separated into two main sections. The first section includes identification of vaccine targets integrating a broad range of computational methodologies in tight interaction with domain knowledge and given experimental data.

The second section comprises target protein analysis and epitope selection specifically focusing on the type of immune response needed for addressing a particular pathogen, but also taking care of the envisaged formulation and delivery platform to be used. T-cell epitope prediction e.g. is particularly well suited for target protein ranking in the context of DNA vaccines for improving reactivity in a given (MHC specific) patient population. Alternatively, putative B- and T-cell epitopes may be selected for designing peptide vaccines. 

A central aspect in downstream formulation is the use of adjuvants (e.g. in the case of DNA vaccines interleukins may be explicitly coded) to increase immunogenicity, and equally important to direct the immune response towards making use of desired effectors (such as humoral versus cytotoxic immune response). Formulated vaccines in a next step have to be validated in animal models for analyzing efficiency in the in-vivo (challenge) situation, complemented by in-vitro assays e.g. testing neutralizing effects of antibodies and reactivity of T-cell epitopes.

Ideally the various steps are embedded in an integrated data and knowledge management framework. Dedicated knowledge management tools are available for reflecting specific procedural structure and access permissions for following standard operating procedures (SOPs). Data representation and visualization generically support such workflows, which, together with a more detailed view on selected procedures raised here, will be discussed in the following sections.

### pBone/pView concepts and implementation

**Figure 2:**
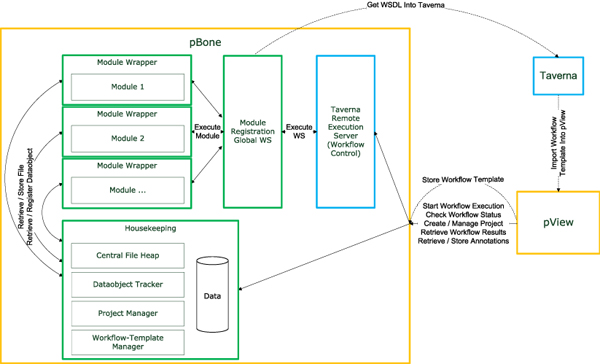
**Schematic overview of the relation between the Taverna Workbench, Taverna Remote Execution Server, pBone and pView**: Workflow templates are generated in the Taverna Workbench and imported into the pBone system via pView. pView then acts as control element for project creation/extension, data import and processing in pBone. Individual components of pBone are set into relation including dependency on Taverna Remote Execution Server for processing of Taverna workflows.

We consider computational workflows as essential for structuring computational vaccinology. Our implementation, pBone/pView, revolves about the idea of modular, pre-defined tasks linked in vaccine (pathogen) specific workflows. Various tools are readily available for supporting computational workflow design, including Taverna, Triana, Kepler and Pegasus [19]. The process (Back) Bone (pBone) takes care of storing and providing process templates and supervising execution of instances of templates on available resources (be it a single server or a cluster). Process View (pView) takes data generated in the workflows, connects (relates) them to what is already available for a particular project and provides a visualization interface, in some aspects comparable to Jalview [20]. The overall concept of pBone and pView is shown in Figure 2.

Taverna provides a graphical user interface to create and execute workflows. For creating pBone workflows the user is provided with access to a set of specialized web services provided by a central pBone server. The pBone web services wrap existing software applications and integrate them into pBone without modification. This web service-centered environment supports data comparison and identification and allows data tracking. 

In the context of pBone a workflow is a set of tasks which depend on their predecessors. Workflows are typically sequential, but branching is possible. If created as atomic as possible, larger workflows can be customized from these generic elements for customizing the flow for specific project requirements. Such workflows furthermore allow researchers to adapt to the fast changing bioinformatics tools environment. 

A central idea of pBone is to gather the data produced by workflows into dedicated projects. A project is defined through a basic set of descriptors such as project name (e.g. EBV), creator, creation date, etc. The amount of project information is kept small on purpose, as pBone and pView are not supporting conventional project management requirements but centrally serve in data generation and subsequent analysis. For a specific project a user may run a customized workflow (or workflow component, e.g. including a newly sequenced EBV isolate). pBone then automatically adds results (multiple alignment) to the associated project and merges these with results of previously run workflow elements for the specific project. pView is designed as a downstream results data exploration environment. A central part of pView is flexible data loading and visualization. The researcher controls a set of specific visualization elements for various data formats at hand. Central visualization modes include protein/nucleotide sequences, sequence alignments, and diverse data compatible with line and block graphs or plain text. 

#### pBone

pBone itself does not execute workflows, these are handled by the Taverna Remote Execution Server. pBone in conjunction with pView provides the remote execution server with entire XML workflow templates including input files required. Each computational task is wrapped into a specific interface for providing additional functionality (Module Wrapper). Wrapper functionality for external software applications is to retrieve data, as well as to register and link new data objects. All available computational modules are registered at the Module Registration Global Web Service. SOAP based web services thereby provide a de-facto standard method for remote access to computational resources.

A wrapper consists of two parts: (a) an XML based configuration file which defines the input required, output generated, and how these are connected with the actual service, and (b) a Java class launching the service and handling pBone specific tasks.

The process of invoking a service on pBone starts by retrieving or storing new data files. To minimize data traffic pBone deposits all incoming and generated data files into a predefined location on the pBone server, the Central File Heap. Each file receives a unique identifier, and a unique md5 hash [21] is calculated. The md5 hash is used to determine if two files are identical to avoid saving files multiple times. A service wrapper has access to the file heap and retrieves files as necessary. The wrapper then builds an execution command for the service and provides input files as needed. The XML configuration file provides the mapping information for the command. This XML file defines which parameters are visible to the user when creating a workflow and how these parameters are mapped on the service. The wrapper then starts the services with a unique home directory for invocation. These unique home directories are necessary to ensure that each generated file can be identified. After the service has finished, the wrapper collects files generated and deposits them into the file heap. The next task of the wrapper is to retrieve data objects saved in the files. This additional level of abstraction allows pBone to identify multiple objects in one file (like a batch of EBV sequences in one FastA file, where each sequence is represented by a data object). Data in the output files are assigned to new data object identifiers, furthermore their relation and the input data object is stored. This connection builds up the hierarchical structure in which objects are grouped.

In addition to the parent-child connection of data objects, pBone is able to determine if two data objects are identical. If a researcher starts two workflows with the same input file (e.g. one for generating a multiple sequence alignment and one for computing secondary structure) within a project, pBone recognizes this redundancy, and the two resulting hierarchical data object structures are merged. This allows users to run multiple workflows with the same initial data at various project stages and still maintain relations between runs. 

pBone introduces three different types for categorizing individual data objects:

1. Sequences are either protein or nucleotide sequences.

2. Descriptors are objects that provide additional information about another object. A descriptor can be, for example, antigenicity, average secondary structure element propensity, or any other sequence feature. A descriptor can be linked to all other data object types by including another descriptor.

3. The Entity type of objects. An example for an Entity is e.g. a multiple sequence alignment as this data set does not provide additional information about a single sequence and is not one single sequence as such. The multiple sequence alignment is to some extent a set of sequences but through the alignment additional information is provided.

The last job of the wrapper is to map output of a service according to the XML configuration file, and return unique identifiers which can be used as input for the next service in the workflow.

pBone and pView store data from different origin into one database. This data diversity naturally complicates development of individual parts of the system. A basic set of interfaces was designed to allow access to individual components within pBone. To build a more robust and flexible database access system pBone and pView use the Hibernate (http://www.hibernate.org) persistence manager. Hibernate hides all database related operations from the user and reduces development time and error rate. By default pBone and pView save data into a relational database. Hibernate takes care of most of the database need of the system and allows simplified change of the backend database server by altering a configuration file. This enables pBone and pView to be deployed in existing environments without the need for a new database infrastructure.

#### pView

**Figure 3:**
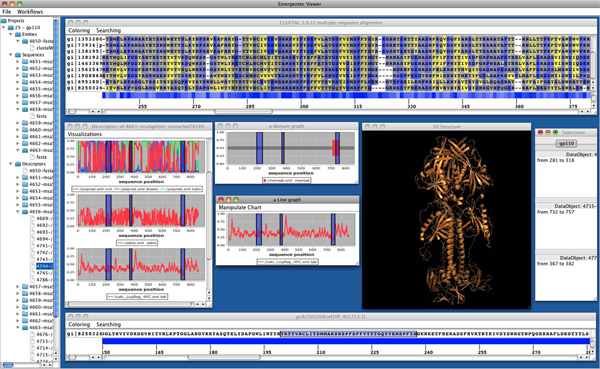
**A typical workflow situation utilizing pView.** On top a multiple sequence alignment is provided. For the sequences various single sequence profiles are given in separate windows further including a 3D model of the protein of interest.

Whereas pBone is responsible for data generation it is the purpose of pView to aid users in analysis of results. Projects are created in pView and workflows are launched through it. To launch a workflow pView makes use of the Taverna Remote Execution Client plugin. After launching a workflow the user is able to add data and their structure to the project. At this point users have connections to newly processed, as well as to existing data. To facilitate data exploration pView provides a visualization front-end allowing to open different content in various formats within pView, additionally providing customized plugins. A visualization plugin consists of two components: The first reads data from a generic source according to its format and represents them in an arbitrary data structure. The second component uses this structure and builds a visual representation. Figure 3 exemplarily displays sequence and structure visualization. pView provides a set of default visualization methods including support for multiple sequence alignment, single sequences, as well as various types of graphs.

Generally, navigation in large data sets is problematic. Similarly, determining the exact position of amino acids in a MSA (multiple sequence alignment), and at the same time in multiple, associated graphs is cumbersome. pView provides a marking system that allows the selection of a range of positions in one data object (e.g. in a sequence alignment) and this selection is then propagated to all associated data objects (e.g. identifying the marked sequence stretch in the corresponding sequence stretch of the  antigenicity profile). The relationship between objects is defined by pBone during workflows execution. Multiple selections are possible and each selection can be saved, making it persistent in the pBone database. Adding a comment to a specific selection allows users to document conclusions drawn from specific data analysis tracks.

pBone and pView shall serve as example workflow implementation for structuring two central procedures in computational vaccinology discussed next: identification of target proteins, followed by identification of antigenic determinants.

### Identification of vaccine targets

Some generic reasoning applies for selecting vaccine targets. Generally, a target should be reasonably conserved across pathogen isolates, while being dissimilar to host factors. Functionalities which are easily lost without gross detrimental effects for the pathogen are not suitable. If a number of sequenced strains is available functional annotation is helpful for assessing essentiality of a particular factor. In this area the interface to bioinformatics procedures for (next generation) sequencing, annotation and data handling is obvious. 

If (unfortunately) no specific information on the proteome is available and consequently proteome-wide antigenicity prediction becomes necessary a ranking of targets may be performed based on suitable classes of cytotoxic and helper T-cell epitopes, lack of cross-reactivity with the host, and conservation of epitopes across strains. Vaccines focusing on humoral (B-cell) responses add some further layers of complexity. Antibody targets are preferred to be accessible (extracellular). If no functional sites (for example protein-protein interaction interfaces of toxins, or protease active sites relevant for pathogenicity) are targeted then the antigen has to be strongly expressed or concentrated on certain patches of the pathogen surface to allow antibody-dependent cell-mediated cytotoxicity (ADCC) or opsonization. Computationally this mingles into prediction of expression (particularly for bacteria) and analysis of respective transcriptomics and/or proteomics data. Numerous bioinformatics routines for functional annotation and prediction of function [22,23], as well as prediction of sub-cellular localization are  useful in this context. Resources such as the pathogen antigen database AntigenDB [24] add value when combined with tools for sequence or HMM-based homology search, furthermore including fold recognition methods for detection of remote homologues. Some aspects of protective antigens have been integrated into dedicated predictors of vaccine targets such as VaxiJen [25].

EBV is a relatively well studied organism; consequently we in the following rely on mapping of known protective epitopes derived from other herpesviruses as well as on literature data concerning relevance of potential target proteins for the viral life-cycle. We included these aspects in a Systems Biology approach to reach further insight into the pathogenicity mechanisms of EBV. The following sections specifically focus on these aspects.

#### Homology mapping of given B-cell epitopes

The aim of B-cell epitope prediction in vaccine design is to identify regions which should be targeted, or, equally important, avoided. The latter include, among others, immunodominant and/or highly variable regions which can act as decoy epitopes (diverting immune responses from more effective targets), and which may provide little cross-protection between isolates. Selected epitopes ideally confer to an area on the protein relevant for function. Antibodies targeted at such sites are referred to as neutralizing, although neutralization of a pathogen may also result from other effects as ADCC, being more dependent on antigen density on a surface rather than on a specific functional site.

B-cell epitopes (antibody binding sites) are intrinsically less stringently defined than T-cell epitopes. Nevertheless, due to their relevance in immunology numerous *in-silico* methods for their prediction have been published. Many of these focus on the prediction of linear B-cell epitopes, some on discontinuous epitopes, and very few attempt to build models for neutralizing epitopes [6,26]. The latter are usually considered ideal targets for vaccines, since the simple binding process may already lead to inactivation of the pathogen or key pathogenicity functionalities. Due to the scarcity of such prediction systems, the availability of utility data, and overall interest in neutralizing immune responses, we have equipped our B-cell epitope discovery workflow with an epitope homology mapping process. This process makes use of already known epitopes to discover new candidates by using information about experimentally validated epitopes and homology (or remote homology between proteins from the whole pathogen family). 

The 31,844 Herpesviridae-associated sequences downloaded from the NCBI formed the basis for a protein homology network. Position specific iterated BLAST (PSI-BLAST) [27] was used to add edges (relations) between proteins based on their degree of homology. For each of the 31,844 sequences three iterations against the uniref90-database [28] were performed to generate a PSSM (position specific scoring matrix) which was used to perform a single BLAST search against the Herpesviridae sequence set. The resulting hits from the final BLAST search were considered as homologues of the initial query protein. For each resulting hit holding an expect value below 10^-20^ an edge between query protein and hit protein was added to the graph. Additional information such as the alignment, expect-value and alignment score were stored as edge attributes. Applying this procedure for all 31,844 Herpesviridae-associated proteins resulted in a weighted, directed graph. This graph does not only connect obviously homologous proteins, but also allows inferring remote homologies. 

Epitopes retrieved from the IEDB were filtered in a manner such that the resulting set of epitopes fulfilled the following four criteria: 1) B-cell epitopes of the type ‘continuous’, 2) source organism must not be EBV, 3) at least one association with a positive assay, 4) antibody binding must result in biological activity. Remaining epitopes were added to the homology graph as vertices and connected to the vertex representing their source protein. Few epitopes could not be directly connected because their source protein was not in the initial set of herpesviridae proteins. In these cases a single BLAST search against the initial set of herpesviridae proteins was conducted to identify the best substitution for the original protein source sequence. The resulting directed graph connects the set of selected B-cell epitopes with their source proteins which are connected to their homologs. Therefore, based on a given epitope this procedure allows identification of proteins which contain a region homologous to the original linear epitope region. The epitope protein homology graph was used to map any of the given epitopes on any of the given EBV sequences. Therefore all possible paths up to a length of three between any of the epitopes and any of the EBV sequences were explored and evaluated by the following criteria: 1) length of the path, 2) quality of the alignment, 3) is the region of the linear epitope within the alignment, and 4) conservation of the linear epitope region.

**Figure 4:**
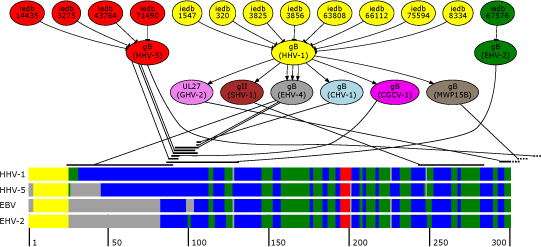
**Presented is an overview of the epitope mapping process.** The upper part depicts a subgraph comprising shortest paths between known epitopes and EBV gp110. The lower part of the figure shows the first 300 positions of a multiple secondary structure alignment of homologous envelope glycoproteins of EBV, HHV-5, HHV-1 and EHV-2. To improve readability secondary structures are color coded (helical areas in red, beta sheets in green, coils in blue, signal peptides in yellow and gaps in grey). The black strands above the multiple alignment mark possible mapping positions with respect to their position on the gp110 protein of EBV which are connected to their predecessor in the shortest path.

After evaluation of the four criteria the highest scoring path was selected and further evaluated by secondary structure comparison and domain alignment between the affected proteins. Figure 4 gives an overview of selected shortest paths between known epitopes and the EBV protein gp110. 

The major advantage of this method compared to PSI-BLAST alone is that the generated PSSM is otherwise centered between identified proteins, possibly posing a problem for borderline homology. Starting from individual identified proteins may therefore yield different PSSMs from homology space thereby increasing sensitivity.

#### Explorative data representation

**Figure 5:**
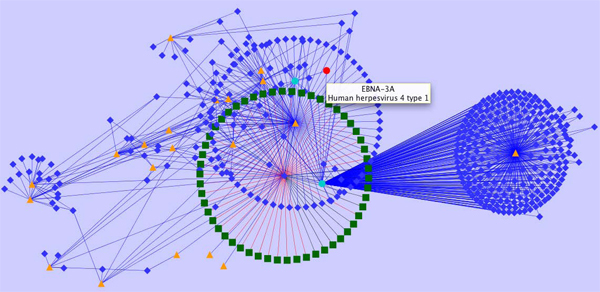
**Representation of heterogeneous data in a network context.** Provided is a subgraph encoding information available for EBV homology data enriched by IEDB object types, relationships and content. Red nodes represent EBV proteins from completely sequenced proteomes which are linked to IEDB data. Turquoise nodes represent proteins listed in the IEDB, orange triangles represent scientific publications, blue diamonds represent peptide epitopes, and green diamonds encode experimental assays.

Analysis of complex data of multiple object types and their relations require viewers capable of representation based on a different paradigm. A general trend is representation of complex biological and associated data in the form of networks. To some level similar to representations of MindMaps [29] context (edge) arranged content (vertices associated with data) allows display of diverse object types such as publications, proteins, peptides or validation data using specific vertex classes brought into relation via specific edge classes. Figure 5 shows an example derived for EBV data. 

The graph given in Figure 5 visualizes a subgraph of selected IEDB content and relations. Utilizing such a representation significantly supports the identification of epitope dependencies, their source organisms, and associated experimental validation status. As an extension to the IEDB the graph links protein variants given in IEDB to a homology network of sequenced herpes viruses. Sequences can thus be linked in a straightforward way by bioinformatics means, further enriched by biological information (epitopes, neutralization status, etc.) and experimental records (assay records, etc.). Various utilities exist for generating such network views including CytoScape [30] which also provides various layout options.  

#### Systems biology

The concept of systems biology is intrinsically biological with some different technological interpretations attached. The basic idea is to view not single, isolated biological objects like transcripts and proteins, but to take their dependencies into account, i.e. interpreting objects in their context. Understanding the host-pathogen interaction allows to shed light on the molecular mechanism connecting infection and disease, being at the core of designing effective vaccines. In the context of EBV this is particularly interesting due to the association with numerous malignancies. The basic idea of using Systems Biology in target selection is to identify essential elements of viral pathogenicity and to select most crucial pathogen components as targets. In the course of this process other issues become relevant, like the use of druggable host (or pathogen) proteins amenable to established medical regimen suitable for auxiliary treatment along therapeutic vaccines.

To obtain a basic set of experimentally determined EBV-EBV and EBV-human protein interaction, we extracted protein interaction data provided by Fossum et al. [31] and Calderwood et al. [32]. These given records were further enriched by interaction data specified in the virusMINT database [33]. While physical interactions are of high value, more complex dependencies may exist between two factors, for example by indirect interaction or genetic co-regulation. For representing such data human genes differentially regulated in the course of EBV primary and re-infection presented by Chen et.al (the Meta-A dataset) [34] were connected using shortest paths utilizing the human proteome interaction network omicsNET [35]. This resulting subgraph was merged with the EBV-EBV and EBV-human subgraphs as noted above, and further enriched by direct omicsNET connections between the two sets of proteins. Further protein-protein interactions between human components given in the graph were added if such an interaction was supported by entries in at least one of four commonly used databases (namely Ophid [36], IntAct [37], BioGrid [38] and Reactome [39]).

**Figure 6:**
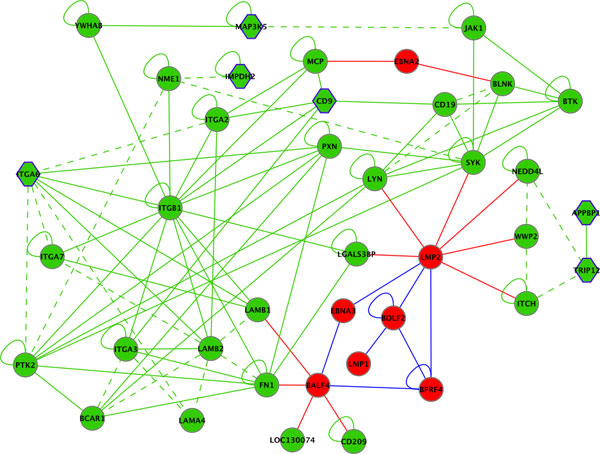
**Selection of vertices and edges from the EBV-human interaction graph centered around differentially regulated CD9.** EBV proteins are shown in red, human proteins in green. Solid lines indicate physical interactions, dashed lines omicsNET connections. Red, blue and green edges indicate EBV-EBV, EBV-human and human-human interactions, respectively. Human genes significantly differentially regulated upon infection/reactivation are shown as hexagon.

A subgraph was exemplarily selected to demonstrate the merits of such integrated interpretation for understanding viral function and subsequently for hypothesis generation supporting target selection. Figure 6 shows specific interaction types and selected protein products of differentially expressed genes in the neighborhood of CD9, a cofactor of CD21 which is the EBV receptor on B-cells [40].

For the generation of this subgraph vertices directly connected to CD9, as well as next neighbors were selected. This subset represents two EBV proteins for which all connected human factors and EBV proteins were added, further enriched by proteins differentially regulated and directly connected.

#### Generation of homology models

For B-cell epitope prediction within this EBV example two proteins were exemplarily chosen to demonstrate a bioinformatics sample process and relevant biological rationale. BALF4 (gp110) and BLLF1 (gp350/gp220) are both essential, surface accessible and well studied components of infectious EBV [41-43]. For both proteins at least partial 3D structures are available in the PDB. gp220 is a splice variant of gp350 [44] lacking a long, according to prediction, strongly glycosylated and intrinsically disordered region being rich in repeats.

Unfortunately these structures are incomplete, primarily lacking some short loops or otherwise unresolved residues, or are non-identical to the sequence of the reference virus used in this study (EBV type 1). While a certain degree of variability will usually not lead to a substantially altered structure it is recommended to visually inspect where changes occur within a 3D context, and also to confirm via homology modeling if these changes might interfere with the structure. In the case of missing loops adding the sequences in structure modeling at least provides an idea which regions may be less accessible by antibodies, or which loops are structurally favorable to use as continuous B-cell epitopes. 

Model templates applied for the EBV example made use of the PDB entries 3FVC and 2H6O for gp110 and gp350, respectively. While 2H6O directly served as template for monomeric gp350, gp110 presumably forms trimers [43,45]. As multimerization of proteins massively alters accessibility of protein domains the likely quaternary structure was retrieved through the PQS interface of PDBe (also part of wwPDB) [46]. Homology modeling was then based on this trimer.

**Figure 7:**
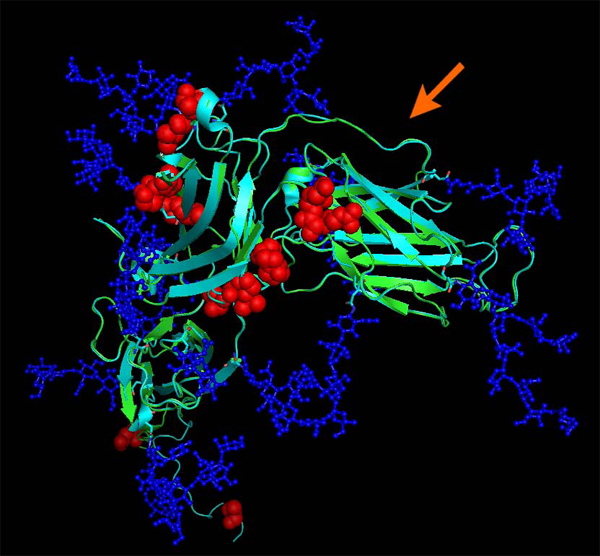
**Structural alignment of gp350 (PDB entry 2H6O, turquoise) and EBV type 1 gp350 N-terminal domain model (green).** Mutations differentiating the two proteins are highlighted by red spheres. Glycosylations (which are part of the 2H6O structure) are drawn in blue and indicate which residues may not be directly accessible to antibodies. Mutations are located outside the CD21 interacting region which is non-glycosylated and has been implicated in neutralizing immunity. The arrow indicates the CD21/gp350 interface.

**Figure 8:**
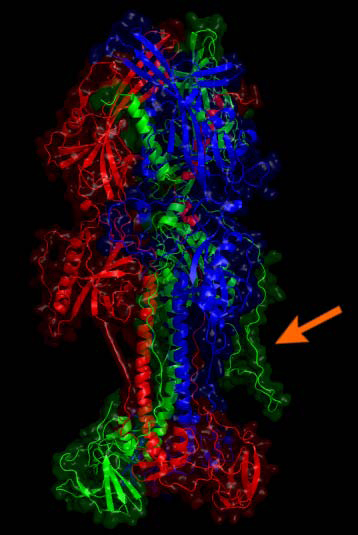
**The optimized model for the EBV type 1 gp110 protein is given.** Monomers are drawn in green, red and blue. The arrow indicates one of the large coils added by homology modeling. The lower part is close to the viral membrane while the stem and head extend into the solvent and are free for molecular interaction. Potential glycosylations were not further considered.

Figure 7 shows the structural alignment of 2H6O serving as template for gp350 superimposed with the modeled structure of EBV type 1. The alignment reveals overall very little dissonance between the structures. Glycosylation was taken from the original structure. Figure 8 shows the optimized trimer model of gp110. One of the added loops is particularly extended (57 residues) and presumably different from the proposed models. Yet it can be beneficial to visualize the approximate extent of a loop for manual epitope selection.

### Identification of antigenic determinants

#### Prediction of B-cell epitopes

We applied various methods for B-cell epitope prediction involving individual sequences or multiple sequence alignments. Sequences for multiple alignments were manually selected based on homology. In general selection from multiple species (rather than multiple isolates) is possible but certainly carries substantial risks. Different viruses, in some cases even isolates of the same virus, can alter tissue tropism and may therefore show characteristic changes in their surface glycoproteins. Those changes may erroneously be interpreted as variability connected to low relevance. To alleviate this problem for our example scenario, isolate-specific EBV sequences and those of closely related viruses (Callitrichine herpesvirus 3 and Pongine herpesviruses 1-3) were aligned to gp110. In the case of gp350 sequences from Cercopithicine herpesvirus 15 and Macacine herpesvirus 4 were added. All four mentioned non-EBV viruses belong to the genus Lymphocryptovirus and can therefore be considered as close relatives of EBV.

**Figure 9:**
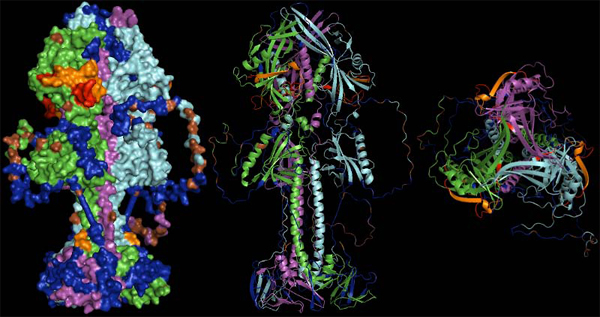
**Representation of the gp110 putative trimer surface and secondary structure cartoons in lateral and top view (left to right).** Monomers are drawn in green, violet and cyan. Regions covered by predicted, potentially neutralizing epitiopes are shown in blue, residues predicted to be glycosylated are given in brown. Areas coded in red were experimentally shown to be neutralizing in homologous proteins of other herpesviruses, while areas coded in orange were additionally predicted as epitopes. The orange spot at the stem of the molecule indicates the terminus of a neutralizing epitope close to the N-terminus of the protein (unfortunately only partially resolved in the structure model).

B-cell epitopes aimed at blocking virus entry or inhibition of viral functions responsible for pathogenesis were then predicted by semi-automatically creating a consensus table comprising results of all applied methods represented on the level of the consensus sequence. A graphical representation of epitope areas for gp110 is given in Figure 9.

Aim was the selection of peptides of at most 15 amino acids in length. For considering a particular protein region as a candidate for protective immune responses (reflecting functional importance as well as antigenicity) at least one of two methods implicated for functional relevance of a linear segment was required to produce a positive prediction in the region, augmented and further localized by at least two different B-cell epitope prediction methods. In addition, surface accessibility, accessibility changes through putative multimerization, and variability were taken into consideration. Briefly, peptides should be well accessible as measured by side chain solvent accessibility determined either from the 3D model where available, or by sequence-based prediction. In addition multimerization should not (or only marginally) alter solvent accessibility of candidate sites on monomers, as this would indicate potential hindrance for accessibility to antibodies. Effects of multimer formation were only assessed for gp110 within the area where the experimental 3D structure was available, as no multimer models are available for gp350. Sequence variability determined from multiple alignments was integrated by shifting peptides where possible to foster a (hypothetical) broadly effective vaccine.

In standard vaccine development settings all available data including experimentally mapped peptides would be integrated to enable an expert in the field to conclude on optimal candidates. In computational vaccinology one validation approach is against experimental sources. In our example validation of predictions were done against IEDB data. In the case of EBV gp110 no neutralizing epitopes have been described so far, which may be astonishing as this protein is among the most conserved sequences of herpesviruses, and resembles an essential component of the attachment/membrane fusion mechanism [47]. Neutralizing epitopes have in contrast been described in HHV-1, HHV-5 and EHV-2 homologues, and these were mapped to gp110. While these mapped epitopes may be neutralizing based on blocking function there is of course also the possibility that indicated regions are neutralizing through a mechanism requiring high surface density of the target protein, e.g. opsonization with complement components. Also, dominance of gp110 and gp350 homologues in cellular attachment can be different even among closely related herpesviruses [48]. While experimental clarification is pending we consider the gp110 peptides identified by homology mapping as being part of neutralizing epitopes as valid assumption. Neutralizing gp350 epitopes were published by Urquiza et al. [42], consequently validation is straightforward.

Over the entire sequence of gp110 19 non-overlapping peptides of 15 residues length at maximum were selected. Seven of those were excluded because of overall low accessibility (or specifically low accessibility of more than 30% of the peptide sequence). Of the remaining 12 candidates, two substantially overlapped with protective peptides as mapped onto gp110. Another mapped sequence reported as neutralizing peptide was missed, however, the immunogenicity of this particular peptide is unclear as the indicated region should be inaccessible according to MEMSAT3 prediction and UniProt [49] entries. One of the likely neutralizing epitopes is in part affected by multimerization but was accepted after manual inspection of the crystal structure (serving as example for the importance of manual results integration and analysis).

From a biological but also from a vaccine design perspective it makes sense to target amino acid repeats, as these may carry relevant functionality and allow to cover extended areas of a target using a single peptide for immunization. Among the two methods applied for detection of internal repeats, RADAR presented by Heger and Holm [50] identified five repeats, and HHrepID derived by Biegert and Söding [51] identified four repeats. Results of these procedures are presented in Table 2. 

**Table 2:** Repeats identified by RADAR.

start	stop	peptide	variant
**521**	**538**	**TSPTPAVTTPTPNATSPT**	1
**542**	**559**	**T**T**PTP**NA**T**S**PT**LGK**TSPT**	2
**563**	**580**	**T**T**PTP**NA**T**S**PT**LGK**TSPT**	2
**584**	**601**	**T**T**PTP**NA**T**S**PT**LGK**TSPT**	2
**605**	**621**	**T**T**PTP**NA**T**G**PT**VGE**TSP**Q	3

Table 2 lists residues dissimilar to peptide sequence variant (1) in bold, identical repeats are considered as one variant.

Manual analysis of the repeat regions showed that the five repeats with three variants, each of length 18, strongly overlapped with a 12-mer repeat which exists in only two variants and also occurred a sixth time. The specific motif of this repeat is ‘VTTPTPNAT[SG]PT’. Presumably this shorter variant has been missed due to maximizing repeat length rather than number of occurrences. This repeat is the most prevalent and invariant motif identified, and strongly enriched in six copies found between residues 512 and 614 of gp350, while lacking in the alternative splice product gp220 (deletion of residues 502-698) [52]. Five of these repeats are present as ‘VTTPTPNATSPT’, which was subsequently included into the list of candidate B-cell epitopes as this region is encompassed by good solvent-accessibility, albeit also N-glycosylation might appear. Only one of these copies (residues 561-572) is associated with a predicted, putatively neutralizing epitope, yet repeats are inherently interesting targets as argued above. The prediction of neutralization in the vicinity of one of these therefore further supported the selection of the minimal motif. Interestingly, Urquiza et al. [42] summarized reports of monoclonal antibodies mapping to the given (or closely associated) repeats, apparently confirming presented considerations. 

Table 3 lists some commonly used amino acid-focused quality metrics for B-cell epitope prediction, while Table 4 focuses on validation on a peptide basis, assuming that substantial overlap or sub-selections of neutralizing epitopes can have the same effect as the entire/exact epitope sequence. 

**Table 3:** Metrics for estimating the quality of neutralising epitope prediction.

protein	baseline	TP	FP	TN	FN	sensitivity	specificity	accuracy	precision	MCC
gp110	5.72%	26	141	667	23	0.53	0.83	0.81	0.16	0.21
gp350 (no repeat)	8.05%	47	224	615	26	0.64	0.73	0.73	0.17	0.22
gp350 (single repeat)	8.05%	59	224	615	14	0.81	0.73	0.74	0.21	0.32
gp350	12.35%	95	224	576	17	0.85	0.72	0.74	0.3	0.39

Table 3 provides TP (True Positives), FP (False Positives), TN (True Negatives), FN (False Negatives), MCC (Matthews correlation coefficient) for selected sequences. The baseline value corresponds to the percentage of residues belonging to neutralizing epitopes. For gp350 tree entries have been added, depending whether a single copy of ‘VTTPTPNATSPT’ is interpreted as False Negatives (FN), namely i) no repeats are considered as predicted, ii) a single repeat is accepted as predicted, or iii) all four copies are accepted as predicted (default).

**Table 4:** Peptide coverage of neutralizing epitopes.

protein	baseline	number of peptides selected	percentage (%) of protein covered	number of potentially neutralizing peptides selected	coverage of known neutralizing epitopes
gp110	5.72%	12	19.49%	2	2/3 = 66%
gp350	12.35%	20	35.17%	5	4/4 = 100%

Table 4 gives the number of selected peptides (and percentage of entire protein covered by these) in combination with the percentage of residues belonging to neutralizing epitopes and coverage of known neutralizing epitopes as an indicator of selection effectiveness for gp110 and gp350. A definition of the baseline is given in Table 3.

To account for the effects of repeat peptides where a single peptide covers multiple sites three different results were included in Table 3. In the first approach selection of the repeat is neglected, interpreting constituent residues of a single occurrence as false negatives. In the second row only one repeat is considered predicted, the others are considered true negatives. In the third row all repeats are considered epitopes and predicted as such (true positives). While the latter is probably most consistent with reality it biases validation as a single peptide drastically increases the number of true positives. It is also obvious that neglecting the repeat strongly reduces sensitivity of predictions. Based on single amino acid evaluation, accuracy and specificity are good while sensitivity could be improved. This impression is alleviated, however, by considering entire peptides. Table 4 shows that in gp110 two out of three, and in gp350 four out of four neutralizing epitopes were re-discovered, where only one of the four is the repeat peptide. In the case of gp350 validation is found to be less biased than for gp110, as epitopes accepted as neutralizing were validated by Urquiza et al. [42] through peptide immunization. It is interesting to know that in this validation more peptides were indicated by cell binding assays than in subsequent neutralization experiments. These peptides carry the essential element of immunogenicity and cross-neutralization with a native antigen rather than the potential to be neutralizing only in a conformational context. 

#### Prediction of T-cell epitopes and variability of T-cell antigenicity

For the prediction of T-cell epitopes also an exemplary approach is presented below, focusing on a few HLA alleles of particular relevance. EBV is one of the causative agents of the most prevalent childhood cancer in equatorial Africa, namely endemic Burkitt's lymphoma (eBL) [53]. This in mind we selected human haplotypes presumably prevalent in the region as these should in consequence be of particular importance for a vaccine and utilized publicly available sequence based T-cell epitope predictors to identify potential epitopes on EBV antigens. In contrast to molecular dynamics based predictions these classifiers can be scaled efficiently to screen entire proteomes against a multitude of MHC alleles for potential T-cell epitopes [54].

#### Selection of suitable MHC/HLA alleles

Dominant HLA alleles were determined by gathering frequently occurring class I and II alleles from a publicly accessible database of allele frequencies for relevant countries [55]. HLA alleles were ranked by a simple score formed by the product of the number of countries they were reported in at minimum levels (usually >= 2%) and median prevalence within those countries. Class I alleles were further analyzed for population coverage by Population Coverage Calculation, a tool provided in conjunction with the IEDB [56]. In addition, MHC supertypes were determined for the highest ranking alleles based on a classification table provided by Sidney et.al [57]. Table 5 shows HLA alleles and their supertypes in order of putative relevance for a vaccine in equatorial Africa.

**Table 5:** HLA alleles particularly indicated for coverage of ethnicities in selected equatorial African countries.

HLA allele	HLA supertype	# of countries	prevalence	score	Cumulative population coverage
B5802	B58	2	10.3	20.6	12.34
B1503	B27	2	7.4	14.8	22.56
B5301	B07	3	4.9	14.7	31.93
B4901	Unclassified	3	4.6	13.8	35.77
B4201	B07	2	6	12	42.23
B4501	B44	3	3.8	11.4	47.41
B1510	B27	2	4.25	8.5	50.57
B1402	B27	2	3.95	7.9	52.84
B3501	B07	3	2.2	6.6	55.8
B8101	B07	2	2.6	5.2	58.49
B5703	B58	2	2.5	5	59.5
B5801	B58	2	2.3	4.6	64.57
A7401	A03	2	2.1	4.2	66.35
B0702	B07	2	1.9	3.8	68.49
B4101	B44	1	2.7	2.7	69.35
A2301	A24	1	2.5	2.5	70.97

Table 5 lists allele data for equatorial Africa as obtained from a publicly accessible allele frequency database. Overall frequencies are biased towards HLA-B alleles. ‘Countries’ indicates the number of countries with a substantial sub-population, ‘Prevalence’ gives the median of allele prevalence in available datasets, ‘Score’ provides the product of Countries and Prevalence, ‘Cumulative Population Coverage’ resembles the cumulative average coverage of  populations in Kenya, Uganda and Rwanda by adding individual alleles in order of their rank.

Regarding genetic immunization (DNA vaccines) the selection of antigens combining epitopes for as many MHC/HLA types as possible is feasible. For peptide vaccines in contrast this constraint does not apply as peptides from multiple proteins can be combined with as much or little effort as for a single protein. We exemplify the analysis for one specific protein and selected epitopes for multiple HLA alleles. For that purpose EBV specific T-cell epitopes were retrieved from IEDB and allele coverage was analyzed. Among six proteins carrying  identified class I T-cell determinants the protein LMP2A showed the broadest coverage of alleles, providing epitopes for HLA-A*2301 (A24), HLA-B*3501 (B07) and HLA-B*1402 (B27).

The possibility of using an unmodified LMP2A DNA vaccine is set aside because utilizing the entire, unmodified LMP2A would likely be detrimental, as this protein resembled a cytoproliferative factor potentially involved in the development of lymphomas and has been associated with autoimmune disorders [58]. For HLA-A*2301 (A24) and HLA-B*3501 (B07) netMHC provides artificial neural network (ANN) predictors trained on allele specific data. Predictions for HLA-B*1402 (B27) can be done via NetMHCpan which implements a pan-allele approach extrapolating ligand specificities from closely related alleles with defined preference.

For MHC class II a few alleles can be found in several different countries, some of them at high frequencies (data not shown). Selected class II alleles include DRB1*1101, DRB1*1102, DRB1*1503, DRB1*0301 and DRB1*1302. Generally, but in particularly for Uganda IEDB tools suggest relatively low population coverage for this allele combination (overall 14.89%). DRB1*1101, DRB1*0301 and DRB1*1302 ligands were predicted using NetMHCII, DRB1*1102, and ligands for DRB1*1503 were derived by applying NetMHCIIpan.

#### Visualization of T-cell antigenicity

T-cell epitopes are continuous fragments (peptides) interspersed throughout a protein sequence. In cases where regions of a protein are to be selected for formulation of a vaccine, independent whether recombinant, DNA subunit, or peptide vaccine, conservancy should be considered. Even small changes in sequence may lead to drastic changes in T-cell antigenicity. Reasons include reduced ligand affinity by alteration of essential anchor residues, decreased proteasomal processing, decreased TAP transport rate, or increased similarity to self antigens. There are different ways to approach these issues. For the workflow discussed here we show a supportive method for manual analysis as well as an automated approach: ‘Conservancy Constrained T-cell Epitope Clusters’ or CCTECs. 

While it is possible and sensible to restrict analysis to fully conserved clusters most pathogens exhibit at least some degree of variability even in functionally essential regions. To avoid exclusion of regions comprising changes we removed only putative ligands where mutants / sequence variants show drastically decreased MHC affinity. In the biological context this means accepting the possibility that T-cell populations may not be efficiently cross-reactive among all variants. Selection of the actual immunogen for formulation can be based on most prevalent peptides, clinically most relevant pathogen isolates, or variants most likely cross-reactive with as many other variants as possible. The latter is difficult to assess as no clear metric has been defined so far, but may be approached using standard substitution matrices such as the BLOSUM series [59,60]. As an alternative and thus alleviating the problem, sets of epitopes can be used for vaccine development. In practice this can be realized by using peptides or possibly short DNA constructs. Epitope mixtures can either be applied in a single, combined formulation of peptides, or in a heterologous vaccine schedule. Either way, using a formulation of mixtures should support the development of cross-reactive T-cell populations while using a heterologous schedule may be more effective in that respect, as only cross-reactive populations are stimulated upon boosting with homologous epitopes distinct from the immune primer.

**Figure 10:**
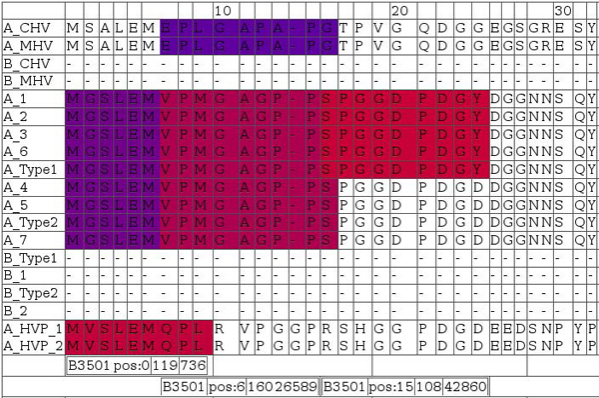
**A snapshot for visualizing T-cell antigenicity of the N-terminal LMP2A.** Data from numerous prediction methods were integrated and visualized in form of an HTML table using a Perl framework. Rows contain aligned EBV sequences; colors indicate degree of antigenicity for a particular allele. The snapshot was selected for three spots of potential HLA-B3501 antigenicity. Bars below the alignments indicate (in this order) allele, start position of the ligand, as well as minimum and maximum IC50 in nM of nanomer peptides. Red indicates high affinity ligands (IC50 in nM around 1), blue indicates low affinity ligands (IC50 in nM around 500).

Manual inspection of data can be insightful in this context, and we developed a simple system for visualization of heterogeneous T-cell epitope prediction results. The software framework integrates output from class I (NetMHC, NetMHCpan) and II (NetMHCII, NetMHCIIpan) ligand, cytotoxic T-cell epitope (NetCTL), as well as proteasomal cleavage site (NetChop) prediction with multiple sequence alignments into a single data source, as exemplarily shown in Figure 10.

Sequence alignments provided in Figure 10 include LMP2-A and -B isoforms denoted by leading 'A_' and 'B_', respectively. Homologous A and B isoforms of Cercopithicine herpesvirus_15 (CHV), Cercopithecine herpesvirus 12 (HVP) and Macacine herpesvirus 4 (MHV) were also included to assess conservation across relatively closely related herpes virus species for delineating indications of constrained sequence evolution. 

#### Conservancy constrained T-cell epitope clusters (CCTEC)

Based on the same data set as used for supporting visual analysis also detection of conservancy constrained T-cell epitope clusters (CCTEC) is performed. The idea is to identify peptides of minimal length which integrate conserved ligands for as many different HLA/MHC alleles as possible. This is one approach for tackling the issue of limited HLA coverage in most peptide vaccines, particularly when compared to subunit or whole pathogen strategies, and can be viewed as an alternative or extension of HLA supertype prediction. HLA supertypes select peptides which cover multiple HLA alleles, while epitope clusters may contain overlapping epitopes. If only class I epitopes are included adequate proteasomal processing should be considered in analysis. Different strategies exist for realization such a concept, depending on whether fixed length peptides are to be selected, only coverage of individual HLA supertypes is considered, or if multiple ligands for each HLA are favorably scored as these will presumably increase the likelihood of peptides to stimulate a relevant immune response. In addition, certain diseases show HLA association, which is to be considered for selecting T-cell epitopes.

The algorithm we describe for this workflow consists of the following components, based on multiply aligned sequences where for each sequence numerous predictive methods are applied:

**1. Identification of conserved antigenic peptides.** Conservation of antigenicity refers to the observation that while sequence can change antigenicity may be conserved. Vice versa, minimal changes may lead to severe alteration of antigenicity. We thus do not require full conservation of aligned (homologous) peptides predicted to be antigenic but consider drop of any of them below 500 nM for class I and II predictions, or below a NetCTL combined score of 0.75 as loss of antigenicity and therefore as an exclusion criterion. While this step does not explicitly respect any classical sequence variability filter such as Shannon entropy there is implicit preference for more conserved regions also on the sequence level, as those are more likely to conserve substantial antigenicity as well. The algorithm considers individually aligned peptides only for the sake of antigenicity conservation, for cluster formation the alignment is treated as a virtual sequence containing conserved regions associated with certain HLA classes.

**2. Identification of clusters.** A window of a width of e.g. 20 amino-acids is moved over the alignment. HLA alleles specific for antigenic, conserved peptides within the windows are registered. A length of 20 is an arbitrarily selected value adequate primarily for peptide vaccines, where synthesis cost and fidelity are of relevance, but much less so for DNA or subunit vaccines. 

**3. Ranking.** 20-mers are ranked by the number of different HLA alleles they serve as ligands. These epitope cluster containing regions are monitored including HLAs covered and degree of HLA coverage (number of ligands per 20-mer).

For each cluster region of interest individual 20-mer peptides constituting the alignment in the region can be accessed and used for formulating a vaccine. Individual peptides can be shorter than 20 residues e.g. based on gaps in the alignment, but usually clusters containing gaps have to be excluded during manual control of output. Results for our implementation of the algorithm can be found in Table 6 where only EBV LMP2A sequences were considered for conservancy analysis although also other organisms are represented in the alignment. EBV LMP2B was also excluded as the N-terminally missing region would otherwise be interpreted as deletion/variability.

**Table 6:** Listing of top-ranked peptides for LMP2A Conservancy Constrained T-cell Epitope Cluster (CCTEC) analysis.

start	stop	HLA count	alleles
177	196	5	A2301(1), B3501(2), B7(1), B27(1), A24(1)
178	197	5	A2301(1), B3501(2), B7(1), B27(1), A24(1)
179	198	5	A2301(1), B3501(2), B7(1), B27(1), A24(1)
230	249	5	A2301(1), B3501(1), B7(1), B27(2), A24(1)
236	255	5	A2301(1), B3501(1), B7(1), B27(4), A24(1)
237	256	5	A2301(1), B3501(1), B7(1), B27(4), A24(1)
243	262	5	A2301(1), B3501(1), B27(1), A24(1), B1402(1)
349	368	5	A2301(1), B1402(1), B7(1), B27(2), A24(1)
350	369	5	A2301(1), B1402(2), B7(1), B27(2), A24(1)
351	370	5	A2301(2), B1402(2), B7(1), B27(2), A24(2)
352	371	5	A2301(2), B1402(2), B7(1), B27(2), A24(2)
353	372	5	A2301(2), B1402(2), B7(1), B27(2), A24(2)
354	373	5	A2301(2), B1402(2), B7(1), B27(2), A24(2)
355	374	5	A2301(2), B1402(2), B7(1), B27(2), A24(2)
120	139	4	A2301(1), B3501(1), B7(1), A24(1)
121	140	4	A2301(1), B3501(1), B7(1), A24(1)
122	141	4	A2301(1), B3501(1), B7(1), A24(1)
123	142	4	A2301(1), B3501(1), B7(1), A24(1)
124	143	4	A2301(1), B3501(1), B7(2), A24(1)
125	144	4	A2301(2), B3501(1), B7(2), A24(2)

Table 6 provides the top 20 peptides from conservancy constrained class I T-cell epitope cluster scan ranked by number of covered HLA alleles. ‘Start’ and ‘Stop’ indicate start and stop residues of the proposed region on the alignment and need therefore not be congruent with positions on the reference sequence. ‘HLA count’ indicates how many different class I HLA alleles and supertypes are covered including the alleles HLA-A*2301, HLA-B*3501, HLA-B*1402 and supertypes B7, B27, A24. Numbers in parenthesis indicate how many ligands were identified within the screening window (20 amino acids) per allele/supertype.

Figure 10 illustrates one of the potential drawbacks or limitations of selecting peptides containing epitope clusters. The third B3501 epitope (alignment positions 16-24 ‘SPGGDPDGY’) is only partially conserved, but if antigenic then this sequence represents a better binder than the other potential ligands. This epitope may therefore dominate the immune response while probably being largely ineffective with respect to almost 50% of isolates. Peptide vaccines can potentially avoid this disadvantage by selecting individual epitopes without constraining to local clusters of high conservancy. Epitope clusters, on the other hand, may have the potential to include several moderately conserved ligands for one allele to alleviate this restriction while reducing the number of peptides to be synthesized.

#### Congruency of prediction and literature

To exemplify validation of T-cell epitope prediction for LMP2, class I T-cell ligands of supertypes relevant for this study were obtained from the IEDB. We consider LMP2 to be an interesting target, both from a vaccine point of view as well as for the sake of demonstration.

Experimentally validated nonamer epitopes for LMP2 extracted from the IEDB are: ‘MGSLEMVPM’ (B07/HLA-B*3501), ‘RRRWRRLTV’ (B27/-), ‘RRWRRLTVC’ (B27/HLA-B*1402) and ‘PYLFWLAAI’ (A24 /HLA-A*2301), where supertype and specific allele are given in parenthesis (if available). Please note that in this context supertype does not refer to supertype ligands in the sense of peptides binding to multiple HLA types within a supertype, but rather that they bind at least one member of the supertype as determined by allele typing.

**Figure 11:**
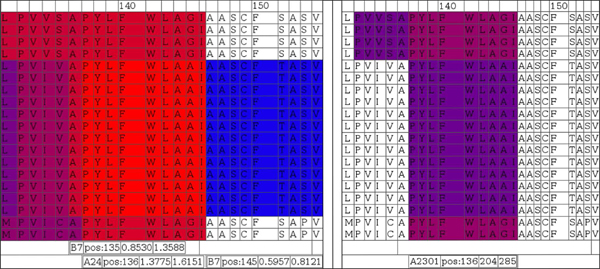
**Color coded display of variability of antigenicity comparing virus isolates in a region associated with an experimentally determined epitope.** NetCTL (left block) and NetMHC (right block) predictions for supertype A24 and specifically HLA-A2301 are shown, respectively. The area represented is centered around known A2301/supertype A24 ligand peptide ‘PYLFWLAA’ starting at alignment position 137. Sequences in the alignments are in the same order as in Figure 10, where the first four and last two sequences are not of EBV origin.

‘MGSLEMVPM’ is fully conserved and is included as the N-terminal peptide in Figure 10. According to prediction this fragment should show moderate affinity to HLA-B*3501 while not being a high affinity binder. ‘RRRWRRLTV’ and ‘RRWRRLTVC’ are strongly overlapping. Both are not considered as good candidates for HLA-B*1402 according to netMHCpan, however netCTL supertype prediction ranks both high with respect to supertype B27, which can be considered as indirect evidence that at least the supertype matches for ‘RRWRRLTVC’. No more detailed typing is included in the IEDB for ‘RRRWRRLTV’. Both peptides are fully conserved even on the sequence level across all human EBV isolates and both LMP2 isoforms. ‘PYLFWLAAI’ is predicted to be a moderate affinity HLA-A*2301 ligand and, following NetCTL, is strongly implicated in supertype A24 antigenicity. Positive NetCTL prediction in this case is mainly based on high ligand affinity and C-terminal processing, and additionally the TAP transport aspect is favorable. A visualization of variability of antigenicity for ‘PYLFWLAAI’ comparing viruses is depicted in Figure 11 (alignment positions 137-145).

## Discussion

### Computational vaccinology workflows

For reducing development efforts a frequently pursued approach is usage of existing software components. For example, pBone and pView rest on Taverna for both, workflow design and execution. Certainly, each software application is designed for a specific task and corresponding specifications, consequently the trade off for adapting software modules has to be kept in mind. However, using frameworks as Taverna renders computational vaccinology workflow design and implementation highly efficient. Similarly, Java offers significant utility libraries.

Web services are the preferred method of communication for pBone and pView, being in widespread use and providing (backed by industry standards) an easy method for accessing computational resources. Restricted efficacy is seen when transferring larger data volumes when e.g. compared to remoting technologies such as RMI or CORBA. For pBone/pView the computational efficacy issue can be neglected because real time responsiveness is not required for such applications. However, large data volumes have to be kept in mind for bioinformatics applications, where Taverna tends to cause problems distributing 10+ MB of data between workflow modules. This problem is rooted in the way web services operate, as the SOA protocol uses XML as a basis for messages. XML parsers often have problems dealing with large files because building the XML data structure is a computationally expensive task. For example, a series of PSI-BLAST runs which were calculated for the homology mapping of the EBV virus resulted in overall 30 GB of data, individual sequences produced PSI-BLAST results of 8 to 10 MB. To combat these data size issues pBone was designed for using file storage, subsequently allowing exchange of file identifiers rather than files as such. A technology currently spreading throughout the Java community is object-relational-mapping. The frequently utilized implementation via Hibernate significantly simplifies the development of database access code. Using Hibernate allows switching the focus to developing application code rather than data persistence code, in turn substantially decreasing overall development time.

Bioinformatics is a fast evolving area commonly featuring short software life cycles, often due to major improvements or development of alternative solutions. When requiring input from multiple sources as is the case in computational vaccinology designing non-monolithic, flexible and adaptable software applications is therefore a logical step. The practical problem faced is that designing such software applications takes more time than creating applications exactly for one narrowly defined task, accenting the importance of a reasonable assessment of flexibility on the one and focus on the other hand. pBone and pView have been made for bioinformatics-rich environments with the analysis of proteins in mind.  However, allowing pBone to make use of an expandable list of bioinformatics tools made it necessary to create a wrapper providing this flexibility.

Multiple sequence alignments and variability are a central part of the visualization concept of pView. At the first glimpse pView looks similar to the popular sequence alignment viewer Jalview. Indeed, both make use of similar concepts such as an MSA centered view, web-services, and inclusion of project support features. The facultative focus on processes with access to a central data repository resembles one of the most significant differences, allowing pBone/pView fast processing of large data volumes. One additional aspect is the ease of adding or editing of (also overlapping) annotations. Annotations are an important collaborative feature, as well as necessary for project controlling. Support for persistence of annotations to a central (pBone) repository was therefore included. To support easy extendibility pView allows addition of new data formats and visualization on a ’plugin’ basis.

### Identification of vaccine targets

Various strategies and predictors exist for selecting vaccine targets and antigenic determinants, also in pathogens less well studied than EBV. Available predictors used in the presented workflow were selected based on superior or at least representative performance judged by state-of-the-art. In many cases comparable alternative methods exist or larger consensus predictions could be included, as is usually the case in a bioinformatics environment. Applied workflows therefore can only represent one possible realization within a larger set of options.

Selection of B-cell targets is strongly synonymous with annotation of genomes, as the assignment of function is usually indicative for relevance e.g. in metabolism, pathogenesis, and possibly conservation across isolates. Expression of proteins only during certain phases of the pathogen life cycle, an aspect sometimes criticized for vaccines generated from cultivated pathogens, is of particular relevance. Similarly, antigen abundance can be tackled to a certain degree utilizing transcriptomics or proteomics data (or alternatively sequencing methods) [61-63]. While level of expression is of high importance for T- as well as B-cell epitopes, understanding of expression variability during pathogen life-cycle such as early and late viral genes can be of importance [64,65]. It is not yet clear whether mimcry or natural immunity should be the aim particularly in chronically infecting pathogens, where immune responses in naive hosts are by definition not particularly successful for clearance of infection. Immuno-diversion or -evasion through specific pathogenicity factors may be more often the problem than lack of effective epitopes or epitope variability.

Homology mapping allows the usage of known epitopes on proteins to predict epitope regions on related (homologous) proteins. Utilizing the concept of homology between biological entities is common practice for various tasks such as gene or protein annotation. Thus the idea of using epitope information from homologous proteins for predicting epitope regions is obvious. Previous work has demonstrated that PSI-BLAST provides an eligible basis particularly for detecting moderate remote homology [66,67].

As in all mapping procedures a few key components exerting major influence on the final outcome have to be respected. The use of the UniRef90 sequence database for generation of the PSI-BLAST homology network reduces runtime and overcomes the problem of over-emphasizing groups of closely related sequences. Using cascading PSI-BLAST may therefore at least in theory be more sensitive to biases resulting in less sensitive searches. In our example the use of only three PSI-BLAST iterations and a stringent cutoff for the final, non-iterative BLAST against the Herpesviridae (e-value <= 10^-20^) ensured that only highly homologous sequences are identified. In contrast to other remote homology detection methods which focus on overall homology at least of domains, our method uses this concept as a first step and then focuses on identified regions containing experimentally validated epitopes. 

Naturally, mapping of protective antigens can be of great value in vaccine design per se. We attempt to make use of as much information as possible by mapping homologous regions of those reported to be involved in protective immune responses. In consequence each significant PSI-BLAST alignment has to be re-evaluated in the context of individual neutralizing peptides. This approach ensures that relevant regions lie within aligned regions, and that the overall secondary structure correlates. Even if simple one-to-one mappings already allow carrying forward experimentally verified data on epitope sequences, the use of multiple mapping steps within a homology network is valuable since it allows propagating information among distantly related proteins at the borderline of PSI-BLAST sensitivity. Care has to be taken, however, to avoid false positive mappings. Alternatively, CSI-BLAST [68] may be applied which was reported to be more sensitive than the widely used NCBI-BLAST.

Sequence areas supported by mapping of multiple epitopes even of different pathogens may indicate that such an area allows targeting several of these pathogens with one peptide when using homologous region if they are at least moderately conserved.

Expectations towards Systems Biology in supporting an understanding of host-pathogen interactions are substantial. While relatively new, this field has already produced encouraging results [69]. Chronically infecting pathogens like EBV are in this context of particular interest due to extensive mechanisms for interfering with the host’s immune system aimed at provoking immune evasion or misdirection [70]. For vaccinology insights into such mechanisms are central for identifying pathogen factors crucial for maintaining viral life-cycle (or even leading to subsequent diseases as malignancies).  

We exemplarily focused on the molecular environment of differentially regulated CD9 on B-cells, a factor associated with the primary EBV receptor CD21, for deriving some of the effects EBV may have on host-cell network organization and function. While EBV can also infect CD21 negative cells (probably through binding to B-cells first) this complement receptor clearly plays a central role in EBV pathogenesis [17]. According to UNIPROT CD21 is expressed on mature B-lymphocytes as well as on some other immunologically relevant cell types and further on pharyngeal epithelial cells. This is of particular interest as the two groups of malignancies which have been speculated to be most likely associated with EBV infection are lymphoid tumours (primarily of B-cell origin) as well as nasopharyngeal carcinomas.

Several observations can be made in a Systems Biology analysis. Among the 48 human factors being in context to EBV in a CD9-centered interaction network 12 are directly targeted by HHV-4 proteins while none of the direct binders is differentially regulated. Probably most apparent is the strong presence of immunologically relevant players (12 of 48). This may not seem astonishing as the network was built around down-regulated CD9, thus likely including factors involved in B-cell regulation, although data on differential regulation were derived from epithelial cells and not B-cells. While CD21 is present in epithelial cells, this specific enrichment of functionality centrally assigned to B-cells is an interesting finding when targeting a virus infecting both cell types. This finding certainly raises the question in how far pathogenicity mechanisms of EBV are specialized for epithelial cells or B-cells, or work similarly in both. There is certainly one major exception, however, as EBV only shows a latent (dormant) status in the B-cell lineage. Concerning interference with homeostatic B-cell regulation, the latency associated antigen LMP2 plays a major role as has been shown previously [71]. LMP2 prevents EBV reactivation in latently infected cells through suppression of SYK and LYN activity [72]. On the other hand this protein bypasses developmental checkpoints allowing immature B-cells to proliferate, an aspect which may be associated with malignancy. Similarly LMP1, a functional CD40 homologue [73], exerts major effects on control of B-cell proliferation [74]. The association with CD209 (DC-SIGN) is not unexpected as this protein is known to bind several envelope viruses including human cytomegalovirus, a relative of EBV [75]. The third observation is the strong enrichment of factors associated with neoplastic disorders. At least eleven out of 48 proteins present in the interaction graph have been explicitly associated with cancer. 

The direct interaction between the latency associated protein EBNA2 and the host factor MCP (CD46) is also of particular interest. MCP acts as a receptor for multiple pathogens including Measles virus and Human Herpesvirus 6 [76,77], the latter being phylogenetically related to EBV. MCP exhibits several immunity associated functionalities, one being a co-stimulatory factor for T-helper cell development which enact at least part of their potential through IL-10 release [78]. Generally, the immune-evasive strategy of EBV appears to rest strongly on IL-10, as the virus itself also codes for an IL-10 homologue (vIL-10; BCRF1) being expressed during the lytic cycle. The immunosuppressive effects mediated through regulatory T-cells during EBV latency may also exert certain side effects pertaining to auto-immune-disease. Hypothetically this may happen through the inability (or decreased efficiency) to fully resolve certain infections in the presence of EBV.

IMPDH2, a rate limiting enzyme of guanine nucleotide synthesis, is consistently down-regulated, much in contrary to what may be expected during oncogenic transformation or increased metabolic needs for virus production. While there is no safe way to generalize findings found for specific tumors, at least in colorectal cancer up-regulation of this protein and associated auto-antibodies were observed [79].  The down-regulation seen may result from anti-viral mechanisms striving to limit the metabolic rate (or may reflect the epithelial origin of the transcriptomics data used). However, IMPDH2 was not reported as consistently differentially regulated in the ‘meta-B’ dataset presented by Chen et al. in both, nasopharyngeal carcinoma and primary effusion lymphoma [34]. Similarly, MAP3K5 up-regulation may be a reaction to viral infection as this protein is thought to be associated with apoptotic death [80]. Interestingly, this is also the case in latently infected cells suggesting viral mechanisms to overcome this apoptotic stimulus. Of relevance in this context is the finding of the down-regulation of the pro-apoptotic factor TRIP12. Down-regulation of APPBP1 (NAE1) is somewhat unexpected as it may suggest attempted cell-cycle arrest also in latently infected cells, while on the other hand avoiding the apoptotic stimulus [81]. However, this may be a tissue specific assumption as apoptosis data was generated in neuronal tissue. Laminin receptor ITGA6 is down-regulated during latent infection but up-regulated during recurrent infection.

The further observation of potential interest derived on the Systems Biology analysis of EBV infection is the interaction between BALF4 (gp110;gB) and human LAMB1 (Laminin subunit beta-1) as well as FN1 (fibronectin). BALF4, and at least its homologue in HHV-1 (UL27;gB), act as essential secondary receptors after initial activity of the primary adhesin (BLLF1 in the case of EBV). UL27 is also essential for initial attachment to host proteoglycans [82]. gp110 is also of specific interest for homology mapping and epitope prediction as this protein is highly conserved in herpesviruses and present in fairly diverse viral species. EBV gp350 (BLLF1) binds to host CD21, and in a second step BALF4 is required for host-membrane fusion [47,82-84]. As LAMB2 and FN1 are both components of the extracellular matrix this may suggest a way to enrich in certain tissues lacking CD21. An alternative interpretation is the potential enrichment of laminins such as LAMB1 (or alternatively fibronectin) in the viral membrane, as these may potentially serve as primary receptors interfacing with gp110. While this may seem far-fetched, LAMB1 and FN1 bind to integrins (receptors) ITGA3 and ITGB1, as well as LAMB1 alone binds to ITGA7. The integrins ITGA3 and ITGB1 also directly interact with CD9 and CD19 suggesting that a hypothetical uptake of laminins or fibronectin in the viral membrane would utilize similar membrane complexes as the classical CD19 assembly. According to UNIPROT annotation LAMB1 is thought to interact with other laminins through coiled coil structures and can be taken up by a high affinity receptor. The reduction of extracellular matrix in cell-cultures may thus be part of the reason why the tissue tropism of EBV seems to be more restricted in-vitro than in-vivo*. A*lso, an increased amount of BALF4 in mature virions can expand tropism to epithelial cells [84,85]. To verify this adhesion hypothesis EBV particle proteomics would be helpful, as host proteins have been shown to be components of virions in Herpes simplex virus 1 [85-87].

The presented interaction network in particular suggests LMP2, EBNA2 and BALF4 (gp110) for inclusion into a list of vaccine targets resulting from the Systems Biology analysis of interfacing to host cellular processes involved in both, immunological response as well as neoplastic disorders.

Methods for selecting candidate B-cell antigens and epitopes for protective immune responses have been significantly put forward in the recent past [25,88]. Particularly with respect to protective epitopes we stress to include functional considerations for B-cell epitope prediction, essentially ranking pure antigenicity as a secondary selection criterion. We consider functionally well accessible sites as preferred targets for stimulating an immune response interfering with relevant pathogen (or other target) functionality. Such considerations primarily pertain to peptide vaccines although it may well be extended to entire domains and select recombinant antigens. The presented approach primarily relies on the classification method previously proposed by Söllner et al. [88], as well as inclusion of potential binding sites in disordered regions [89]. This concept will have to be significantly expanded as new predictors for functional sites [90-92] and annotation resources with respect to target coverage expand [93,94]. From our point of view prediction of B-cell epitopes is far less an issue for one specific epitope prediction routine than a suite of bioinformatics resources for characterizing a candidate protein. Routines applied in this area are naturally in constant flow due to generally short software life-cycles in bioinformatics. Dedicated predictors for continuous as well as discontinuous epitopes are just one element. In the study presented here semi-manual analysis of protectivity yielded encouraging results. Comparison of precision and baseline for amino acid based comparison and particularly coverage of known neutralizing epitopes clearly demonstrate the practical benefit for utilizing such tools in vaccinology. The concept of considering functionally conserved sites is naturally also applicable to T-cell epitopes. In this context it will also be interesting whether furin dependent maturation of EBV gp110 is therapeutically tangible [47]. In the realm of T-cell epitope prediction several successful methods have been published. For this study we selected a number of human HLA alleles relevant for equatorial Africa and integrated predictions performed by several of the CBS tools (netMHC, netCTL, netMHCpan, netChop, netMHCIIpan) into a format amenable for visual analysis of variability of antigenicity. In this context it is worth mentioning that while the difference between antigenicity and immunogenicity of peptides is a highly important concept it is often underappreciated in immunoinformatics practice. Modeling of immunological pathways will likely improve prediction of antigenicity, whereas immunogenicity and immunodominance are decidedly less well understood and represented in models. Nevertheless we see prediction of antigenicity as a reasonable estimate of immunogenicity and immunodominance.

While manual analysis of single sequences for a reduced set of alleles has its merits it is often necessary to screen proteins or entire proteomes for peptides covering multiple alleles. Cluster contained epitopes may not necessarily be immuno-dominant during natural infection but may still provide the merit of broad HLA/MHC coverage. This approach tends to identify supertype binders for several MHC classes or alternatively potentially overlapping epitopes depending on the length of input epitopes and length of the output peptides. We term this concept ‘Conservancy Constrained T-cell Epitope Cluster (CCTEC)’ and understand it as a complement or extension of previously published methods for optimizing vaccine coverage [95]. For the implementation presented here we used fixed width windows leading to ranked peptides of equal length. While this is a straightforward approach it may be preferred to allow peptides within a certain length range, possibly applying a penalty function for particularly long peptides. This would lead to most efficient selections in terms of covering most alleles with as few amino acids to be included as possible. As an additional advantage C-terminal processing of epitopes would be less relevant as at least a fraction of the epitopes would end with the cluster C-terminus thus potentially leading to enhanced availability for MHC loading. Also it may be desirable to weight specific alleles and allele classes differently, for example for achieving optimal coverage of one supertype before searching for additional supertype specificities. One aspect to consider for T-cell epitope clusters is that they are probably best suited for DNA vaccines, as encoded proteins go through the proteasome for which good predictive models such as netChop are available. For peptide and subunit vaccines the lysosomal pathway of proteolysis should apply, so the prediction of dominantly produced peptides ready for cross-presentation [96] may be a hurdle for efficient application of CCTECs in dendritic cell based (T-cell focused) peptide vaccines. In any case, validation of T-cell epitopes, optimally in suitable animal models [97], is of crucial importance due to various difficult to predict factors [98].

## Conclusions

Integration of bioinformatics workflows for target, peptide, or subunit selections, combined with experimental results and overall management of such distributed knowledge are key for enabling efficient and high quality vaccine designs. Integration is of particular relevance for pathogens where experimental setups are complicated or nested, e.g. for determining optimal dosage of adjuvants suited for specific steering of immune responses [99]. We consider a better integration of literature mining results with standard procedures for target selection and decision making as further key area, next to the specific relevance of a multitude of bioinformatics procedures in the context of computational vaccinology [4]. Certainly most of the immunoinformatics modules need further improvement and new models have to be derived e.g. for accessing cross-reactivity between epitopes [100]. The latter is of crucial importance to decide on the number of epitopes necessary to efficiently cover a site with given variability, or to decide whether host or unrelated pathogen epitopes may be cross-reactive. Among the many aspects to be improved, homology mapping should not remain focused on continuous epitopes only, but attempts should be made to map any type of protectivity data. Omics data will be needed including splicing information, as suggested e.g. by opposed effects of human ST5 and particularly implicated for isoform abundance altering viruses such as EBV [101]. In particular a closer link to genome sequencing will be seen in the near future [102].

## Methods

### Data sources and retrieval

All protein sequences associated with the family of Herpesviridae were retrieved from NCBI. As of August 2009 the Herpesviridae family contained 31,844 sequences. 

B-cell epitopes for all herpes viruses were downloaded from the IEDB [103] in XML format (data status as of May 2009). Peptide sequences and additional data such as functional effect of antibodies were extracted from this source. For the purpose of protectivity prediction we consider ‘antibody leading to biological activity’ as sufficiently precise.

Two complete EBV genomes have so far been deposited at the NCBI, namely EBV type 1 (wt) and EBV type 2 (strain AG876), where these assemblies may be reconstituted from multiple isolates [104,105]. In addition to these two complete standard (reference) sequences numerous partial genomes from other viral isolates are available.

### Homology modeling

Homology models were generated using MODELLER version 9v6 [106]. Adequate templates were identified using BLAST searches against RCSB PDB (part of wwPDB) sequences [107]. PDB entries 3FVC and 2H6O for gp110 and gp350, respectively, were identified and used during the modeling process. The sequence available from PDB entry 3FVC and the equivalent part of gp110 are to 99% identical. In the case of gp350 the two sequence parts are to 98% identical. For multi-chain models the modeling setup was based on the data provided in ‘model-multichain.py’. Loops were optimized using the ‘dope’ loop model. For optimization, the functions ‘library_schedule’ (VTFM optimization) and ‘md_level’ were both set to ‘slow’. 

For asparagine and glutamine amide rotamer correction we used NQ-Flipper [108], for visualization of 3D molecular structures we used Pymol [109].

### Prediction of B-cell epitopes

Functional context was assigned by ANCHOR (disorder to order transitions in motif contexts) [89,110], or effective prediction involving protective sites [88]. Methods for prediction of continuous B-cell epitopes comprised COBEpro [111], FBCpred [112] and PCA19 previously reported in association with protectivity prediction [88], complemented by a method referred to as clusters of optimal peptides (COP). Briefly, the proposed protectivity predictions work through machine-learning models based on 10-mer peptides featuring an unweighted total score integrating maximum antigenicity, minimized probability for post-translational modifications, and minimal variability. Instead of using these models the optimized 10-mers can be directly used for determination of clusters. For the COP approach we selected at least the 25 most-optimal peptides per protein and retrieved sequence stretches where selected peptides overlap as most promising areas. Screening for discontinuous epitopes on crystallized or modeled protein domains was done by utilizing DiscoTope [113] and ElliPro [114].

All methods were applied using standard settings for each routine, for COBEpro a minimum cutoff value of 15 was used. For assessment of trans-membrane topology MEMSAT3 [115] and TMHMM2 [116,117] were used, sequence-based solvent accessibility prediction was done applying Sable [118,119]. Protein glycosylation was determined using NetNGlyc and NetOGlyc [120,121]. Presence and boundaries of protein internal repeats were analyzed using RADAR [50], HHrepID [51], and by performing manual inspection. Quality of B-cell epitope predictions was measured using sensitivity, specificity, accuracy and the Matthews correlation coefficient (MCC), a common measure for the quality of binary classification [122,123].

### Prediction of T-cell epitopes

T-cell epitopes were predicted using tools provided by the Center of Biological Sequence analysis, Technical University of Denmark. In particular NetMHC 3.0 [124,125], NetCTL v1.2 [126], NetMHCII v1.1 [127] and NetChop v3.0 [128] were applied. For class I and class II epitopes where no dedicated classifiers were provided ligand affinities as determined by NetMHC, NetMHCpan [129], and NetMHCIIpan [130] were used. 

### pBone/pView relevant software and software libraries

Software platforms and libraries necessary for realizing the technical implementation of the concept of pBone / pView presented in this publication are available in the public domain. Workflow design and execution can be realized using Taverna [16] (http://www.taverna.org.uk/). The Taverna Workbench (http://www.mygrid.org.uk/tools/taverna/taverna-1/taverna-download/) provides an easy to use graphical interface for workflow design, the Taverna Remote Execution Service (http://www.mygrid.org.uk/tools/taverna/associated-tools/taverna-remote-exec ution/) allows to schedule and execute workflows on a dedicated server. The Remote Execution Service provides an easy to use REST webservice interface which can be utilized to manage workflows directly within pView. The module wrapper can be realized using plain Java. Implementation of a common interface ensures that wrapped modules can be registered at the Module Registration Global Webservice. Housekeeping work necessary to retrieve input data and persist output data can be achieved by interacting with the other pBone software components, execution of the wrapped program can be realized using the Java System class which provides functionality to execute system commands in a separated sub-process. The Module Registration Global Web Service can be realized using the Apache Axis (http://ws.apache.org/axis2/) library to expose modules via a webservice, and Apache Tomcat (http://tomcat.apache.org/) to deploy it. Instead of those two components Soaplab [131] (http://soaplab.sourceforge.net/) could be used to expose command-line programs as webservice, however some additional code for required housekeeping functionality would still be necessary. The other pBone software components, such as the Central File Heap or the Project Manager, are straight forward to implement using Java as programming language, the Hibernate framework (http://www.hibernate.org/) for object relational mapping, and MySQL (http://www.mysql.com/) as database management system. However, using well defined interfaces for each component and keeping a modular design in mind is critical for a successful implementation. Based on individual needs the single components can be interconnected using webservices or may be aggregated into a single monolithic component.

The concept of pView may be realized using Java Swing and a conventional model view control architecture. Basically, data generated during workflow execution can be retrieved via the corresponding pBone components, e.g. Central File Heap, Dataobject Tracker and Project Manager, transformed into an adequate data structure using the reader supplied with the corresponding visualization plugin and visualized with the visualization component supplied by the visualization plugin. Arranging those data structures in a graph like data structure eases the implementation of the range selection propagation features. Communication can be realized via webservices using Apache Axis. The BioJava [132] (http://biojava.org/wiki/Main_Page)- and the Phylogenetic Analysis Library [133] (http://www.cebl.auckland.ac.nz/pal-project/) come in handy for reading various file formats. The JFreeChart library (http://www.jfree.org/jfreechart/) provides straight forward ways to realize chart based visualizations. The Java Universal Network/Graph Framework (http://jung.sourceforge.net/) can be used to manage the data structures in a graph like data structure. Instead of implementing pView from scratch it can be realized by extending Jalview [20] (http://www.jalview.org/).

A comprehensive set of workflow modules can be assembled from public domain software. For example the calculation of multiple sequence alignments can be realized using MUSCLE [134]. Memsat3 [115], Sable [118] and Psipred [135] allow to predict trans-membrane domains, solvent accessibility and secondary structure, respectively.

## Competing interests

The authors declare having no competing interests

## Authors’ contributions

BM initiated the study, participated in design and coordination and participated in manuscript preparation. AH and GS designed and implemented the pBone/pView system, AH was responsible for the homology mapping process and was strongly involved in the Systems Biology analysis. JS conceived of the pBone/pView system, designed the study, drafted the manuscript and performed all work related to homology modeling and epitope predictions and was strongly involved in Systems Biology analyses. RF provided data and data processing for Systems Biology analyses. SS contributed to target selection and together with LS critically reviewed the manuscript.

## References

[B1] Hopp TP Retrospective: 12 years of antigenic determinant predictions, and more. Peptide Research 1993 6 183 190 7691280

[B2] Greenbaum JA Kotturi MF Kim Y Oseroff C Vaughan K Salimi N Vita R Ponomarenko J Scheuermann RH Sette A Peters B Pre-existing immunity against swine-origin H1N1 influenza viruses in the general human population. Proceedings of the National Academy of Sciences of the United States of America 2009 106 20365 20370 10.1073/pnas.0911580106 19918065 PMC2777968

[B3] Nielsen M Lundegaard C Blicher T Lamberth K Harndahl M Justesen S Røder G Peters B Sette A Lund O Buus S NetMHCpan, a method for quantitative predictions of peptide binding to any HLA-A and -B locus protein of known sequence. PloS One 2007 2 e796 10.1371/journal.pone.0000796 17726526 PMC1949492

[B4] Flower DR Bioinformatics for Vaccinology 2008 1 Wiley

[B5] Van Regenmortel MHV The rational design of biological complexity: a deceptive metaphor. Proteomics 2007 7 965 975 10.1002/pmic.200600407 17370255

[B6] Yang X Yu X An introduction to epitope prediction methods and software. Reviews in Medical Virology 2009 19 77 96 10.1002/rmv.602 19101924

[B7] Gershburg E Pagano JS Epstein-Barr virus infections: prospects for treatment. The Journal of Antimicrobial Chemotherapy 2005 56 277 281 10.1093/jac/dki240 16006448

[B8] Cohen JI Bollard CM Khanna R Pittaluga S Current understanding of the role of Epstein-Barr virus in lymphomagenesis and therapeutic approaches to EBV-associated lymphomas. Leukemia & Lymphoma 2008 49 Suppl 1 27 34 10.1080/10428190802311417 18821430 PMC2788999

[B9] Lahiri A Das P Chakravortty D Engagement of TLR signaling as adjuvant: towards smarter vaccine and beyond. Vaccine 2008 26 6777 6783 10.1016/j.vaccine.2008.09.045 18835576

[B10] De Gregorio E D'Oro U Wack A Immunology of TLR-independent vaccine adjuvants. Current Opinion in Immunology 2009 21 339 345 10.1016/j.coi.2009.05.003 19493664

[B11] Mutwiri G Gerdts V Lopez M Babiuk LA Innate immunity and new adjuvants. Revue Scientifique Et Technique (International Office of Epizootics) 2007 26 147 156 17633299

[B12] Nickle DC Rolland M Jensen MA Pond SLK Deng W Seligman M Heckerman D Mullins JI Jojic N Coping with viral diversity in HIV vaccine design. PLoS Computational Biology 2007 3 e75 10.1371/journal.pcbi.0030075 17465674 PMC1857809

[B13] Azizi A Diaz-Mitoma F Viral peptide immunogens: current challenges and opportunities. Journal of Peptide Science: An Official Publication of the European Peptide Society 2007 13 776 786 17853502 10.1002/psc.896

[B14] Bijker MS Melief CJM Offringa R van der Burg SH Design and development of synthetic peptide vaccines: past, present and future. Expert Review of Vaccines 2007 6 591 603 10.1586/14760584.6.4.591 17669012

[B15] Purcell AW McCluskey J Rossjohn J More than one reason to rethink the use of peptides in vaccine design. Nature Reviews. Drug Discovery 2007 6 404 414 10.1038/nrd2224 17473845

[B16] Hull D Wolstencroft K Stevens R Goble C Pocock MR Li P Oinn T Taverna: a tool for building and running workflows of services. Nucleic Acids Research 2006 34 W729 732 10.1093/nar/gkl320 16845108 PMC1538887

[B17] Niller HH Wolf H Minarovits J Regulation and dysregulation of Epstein-Barr virus latency: implications for the development of autoimmune diseases. Autoimmunity 2008 41 298 328 10.1080/08916930802024772 18432410

[B18] Pohl D Epstein-Barr virus and multiple sclerosis Journal of the Neurological Sciences 2009 286 62 64 10.1016/j.jns.2009.03.028 19361810

[B19] De Roure D Goble C Software Design for Empowering Scientists. IEEE Software 2009 26 88 95 10.1109/MS.2009.22

[B20] Waterhouse AM Procter JB Martin DMA Clamp M Barton GJ Jalview Version 2--a multiple sequence alignment editor and analysis workbench. Bioinformatics (Oxford, England) 2009 25 1189 1191 10.1093/bioinformatics/btp033 19151095 PMC2672624

[B21] The MD5 Message-Digest Algorithm. http://tools.ietf.org/html/rfc1321

[B22] Fieldhouse RJ Merrill AR Needle in the haystack: structure-based toxin discovery. Trends in Biochemical Sciences 2008 33 546 556 10.1016/j.tibs.2008.08.003 18815047

[B23] Sachdeva G Kumar K Jain P Ramachandran S SPAAN: a software program for prediction of adhesins and adhesin-like proteins using neural networks. Bioinformatics (Oxford, England) 2005 21 483 491 10.1093/bioinformatics/bti028 15374866 PMC7109999

[B24] Ansari HR Flower DR Raghava GPS AntigenDB: an immunoinformatics database of pathogen antigens. Nucleic Acids Research 2009 19820110 10.1093/nar/gkp830 PMC2808902

[B25] Doytchinova IA Flower DR VaxiJen: a server for prediction of protective antigens, tumour antigens and subunit vaccines. BMC Bioinformatics 2007 8 4 10.1186/1471-2105-8-4 17207271 PMC1780059

[B26] Reitmaier R Reitmaier R Review of Immunoinformatic approaches to in-silico B-cell epitope prediction. Nature Precedings 2007

[B27] Altschul SF Madden TL Schäffer AA Zhang J Zhang Z Miller W Lipman DJ Gapped BLAST and PSI-BLAST: a new generation of protein database search programs. Nucleic Acids Research 1997 25 3389 3402 10.1093/nar/25.17.3389 9254694 PMC146917

[B28] Suzek BE Huang H McGarvey P Mazumder R Wu CH UniRef: comprehensive and non-redundant UniProt reference clusters. Bioinformatics (Oxford, England) 2007 23 1282 1288 10.1093/bioinformatics/btm098 17379688

[B29] Buzan T The Mind Map Book 2006 1 BBC Active

[B30] Shannon P Markiel A Ozier O Baliga NS Wang JT Ramage D Amin N Schwikowski B Ideker T Cytoscape: a software environment for integrated models of biomolecular interaction networks. Genome Research 2003 13 2498 2504 10.1101/gr.1239303 14597658 PMC403769

[B31] Fossum E Friedel CC Rajagopala SV Titz B Baiker A Schmidt T Kraus T Stellberger T Rutenberg C Suthram S Bandyopadhyay S Rose D von Brunn A Uhlmann M Zeretzke C Dong Y Boulet H Koegl M Bailer SM Koszinowski U Ideker T Uetz P Zimmer R Haas J Evolutionarily conserved herpesviral protein interaction networks. PLoS Pathogens 2009 5 e1000570 10.1371/journal.ppat.1000570 19730696 PMC2731838

[B32] Calderwood MA Venkatesan K Xing L Chase MR Vazquez A Holthaus AM Ewence AE Li N Hirozane-Kishikawa T Hill DE Vidal M Kieff E Johannsen E Epstein-Barr virus and virus human protein interaction maps. Proceedings of the National Academy of Sciences of the United States of America 2007 104 7606 7611 10.1073/pnas.0702332104 17446270 PMC1863443

[B33] Chatr-aryamontri A Ceol A Peluso D Nardozza A Panni S Sacco F Tinti M Smolyar A Castagnoli L Vidal M Cusick ME Cesareni G VirusMINT: a viral protein interaction database. Nucleic Acids Research 2009 37 D669 673 10.1093/nar/gkn739 18974184 PMC2686573

[B34] Chen X Liang S Zheng W Liao Z Shang T Ma W Meta-analysis of nasopharyngeal carcinoma microarray data explores mechanism of EBV-regulated neoplastic transformation. BMC Genomics 2008 9 322 10.1186/1471-2164-9-322 18605998 PMC2491640

[B35] Bernthaler A Mühlberger I Fechete R Perco P Lukas A Mayer B A dependency graph approach for the analysis of differential gene expression profiles. Molecular BioSystems 2009 19585005 10.1039/b903109j

[B36] Brown KR Jurisica I Online predicted human interaction database. Bioinformatics (Oxford, England) 2005 21 2076 2082 10.1093/bioinformatics/bti273 15657099

[B37] Aranda B Achuthan P Alam-Faruque Y Armean I Bridge A Derow C Feuermann M Ghanbarian AT Kerrien S Khadake J Kerssemakers J Leroy C Menden M Michaut M Montecchi-Palazzi L Neuhauser SN Orchard S Perreau V Roechert B van Eijk K Hermjakob H The IntAct molecular interaction database in 2010. Nucleic Acids Research 2010 38 D525 531 10.1093/nar/gkp878 19850723 PMC2808934

[B38] Stark C Breitkreutz B Reguly T Boucher L Breitkreutz A Tyers M BioGRID: a general repository for interaction datasets. Nucleic Acids Research 2006 34 D535 539 10.1093/nar/gkj109 16381927 PMC1347471

[B39] Vastrik I D'Eustachio P Schmidt E Joshi-Tope G Gopinath G Croft D de Bono B Gillespie M Jassal B Lewis S Matthews L Wu G Birney E Stein L Reactome: a knowledge base of biologic pathways and processes. Genome Biology 2007 8 R39 10.1186/gb-2007-8-3-r39 17367534 PMC1868929

[B40] D'Addario M Ahmad A Morgan A Menezes J Binding of the Epstein-Barr virus major envelope glycoprotein gp350 results in the upregulation of the TNF-alpha gene expression in monocytic cells via NF-kappaB involving PKC, PI3-K and tyrosine kinases. Journal of Molecular Biology 2000 298 765 778 10.1006/jmbi.2000.3717 10801347

[B41] Szakonyi G Klein MG Hannan JP Young KA Ma RZ Asokan R Holers VM Chen XS Structure of the Epstein-Barr virus major envelope glycoprotein. Nature Structural & Molecular Biology 2006 13 996 1001 10.1038/nsmb1161 17072314

[B42] Urquiza M Lopez R Patiño H Rosas JE Patarroyo ME Identification of three gp350/220 regions involved in Epstein-Barr virus invasion of host cells. The Journal of Biological Chemistry 2005 280 35598 35605 10.1074/jbc.M504544200 16087675

[B43] Backovic M Longnecker R Jardetzky TS Structure of a trimeric variant of the Epstein-Barr virus glycoprotein B. Proceedings of the National Academy of Sciences of the United States of America 2009 106 2880 2885 10.1073/pnas.0810530106 19196955 PMC2650359

[B44] Beisel C Tanner J Matsuo T Thorley-Lawson D Kezdy F Kieff E Two major outer envelope glycoproteins of Epstein-Barr virus are encoded by the same gene. Journal of Virology 1985 54 665 674 2987520 10.1128/jvi.54.3.665-674.1985 PMC254850

[B45] Park SJ Seo M Lee SK Lee BJ Membrane binding properties of EBV gp110 C-terminal domain; evidences for structural transition in the membrane environment. Virology 2008 379 181 190 10.1016/j.virol.2008.06.031 18687450

[B46] Henrick K Thornton JM PQS: a protein quaternary structure file server. Trends in Biochemical Sciences 1998 23 358 361 10.1016/S0968-0004(98)01253-5 9787643

[B47] Sorem J Longnecker R Cleavage of Epstein-Barr virus glycoprotein B is required for full function in cell-cell fusion with both epithelial and B cells. The Journal of General Virology 2009 90 591 595 10.1099/vir.0.007237-0 19218203 PMC2768059

[B48] Herold BC Gerber SI Belval BJ Siston AM Shulman N Differences in the susceptibility of herpes simplex virus types 1 and 2 to modified heparin compounds suggest serotype differences in viral entry. Journal of Virology 1996 70 3461 3469 8648678 10.1128/jvi.70.6.3461-3469.1996 PMC190219

[B49] Apweiler R Bairoch A Wu CH Barker WC Boeckmann B Ferro S Gasteiger E Huang H Lopez R Magrane M Martin MJ Natale DA O'Donovan C Redaschi N Yeh LL UniProt: the Universal Protein knowledgebase. Nucleic Acids Research 2004 32 D115 119 10.1093/nar/gkh131 14681372 PMC308865

[B50] Heger A Holm L Rapid automatic detection and alignment of repeats in protein sequences. Proteins 2000 41 224 237 10.1002/1097-0134(20001101)41:2<224::AID-PROT70>3.0.CO;2-Z 10966575

[B51] Biegert A Söding J De novo identification of highly diverged protein repeats by probabilistic consistency. Bioinformatics (Oxford, England) 2008 24 807 814 10.1093/bioinformatics/btn039 18245125

[B52] Baer R Bankier AT Biggin MD Deininger PL Farrell PJ Gibson TJ Hatfull G Hudson GS Satchwell SC Séguin C DNA sequence and expression of the B95-8 Epstein-Barr virus genome. Nature 1984 310 207 211 10.1038/310207a0 6087149

[B53] Chene A Donati D Orem J Mbidde ER Kironde F Wahlgren M Bejarano MT Endemic Burkitt's lymphoma as a polymicrobial disease: new insights on the interaction between Plasmodium falciparum and Epstein-Barr virus. Seminars in Cancer Biology 2009 19 411 420 10.1016/j.semcancer.2009.10.002 19897039

[B54] Tsurui H Takahashi T Prediction of T-cell epitope. Journal of Pharmacological Sciences 2007 105 299 316 10.1254/jphs.CR0070056 18094522

[B55] Middleton D Menchaca L Rood H Komerofsky R New allele frequency database: http://www.allelefrequencies.net Tissue Antigens 2003 61 403 407 10.1034/j.1399-0039.2003.00062.x 12753660

[B56] Bui H Sidney J Dinh K Southwood S Newman MJ Sette A Predicting population coverage of T-cell epitope-based diagnostics and vaccines. BMC Bioinformatics 2006 7 153 10.1186/1471-2105-7-153 16545123 PMC1513259

[B57] Sidney J Peters B Frahm N Brander C Sette A HLA class I supertypes: a revised and updated classification. BMC Immunology 2008 9 1 10.1186/1471-2172-9-1 18211710 PMC2245908

[B58] Swanson-Mungerson M Longnecker R Epstein-Barr virus latent membrane protein 2A and autoimmunity. Trends in Immunology 2007 28 213 218 10.1016/j.it.2007.03.002 17398159

[B59] Henikoff S Henikoff JG Amino acid substitution matrices from protein blocks. Proceedings of the National Academy of Sciences of the United States of America 1992 89 10915 10919 10.1073/pnas.89.22.10915 1438297 PMC50453

[B60] Henikoff S Henikoff JG Performance evaluation of amino acid substitution matrices. Proteins 1993 17 49 61 10.1002/prot.340170108 8234244

[B61] Herbeck JT Wall DP Wernegreen JJ Gene expression level influences amino acid usage, but not codon usage, in the tsetse fly endosymbiont Wigglesworthia. Microbiology 2003 149 2585 2596 10.1099/mic.0.26381-0 12949182

[B62] Roymondal U Das S Sahoo S Predicting gene expression level from relative codon usage bias: an application to Escherichia coli genome. DNA Research: An International Journal for Rapid Publication of Reports on Genes and Genomes 2009 16 13 30 19131380 10.1093/dnares/dsn029 PMC2646356

[B63] Willenbrock H Ussery D Prediction of highly expressed genes in microbes based on chromatin accessibility. BMC Molecular Biology 2007 8 11 10.1186/1471-2199-8-11 17295928 PMC1805505

[B64] Oseroff C Kos F Bui H Peters B Pasquetto V Glenn J Palmore T Sidney J Tscharke DC Bennink JR Southwood S Grey HM Yewdell JW Sette A HLA class I-restricted responses to vaccinia recognize a broad array of proteins mainly involved in virulence and viral gene regulation. Proceedings of the National Academy of Sciences of the United States of America 2005 102 13980 13985 10.1073/pnas.0506768102 16172378 PMC1236582

[B65] Pasquetto V Bui H Giannino R Banh C Mirza F Sidney J Oseroff C Tscharke DC Irvine K Bennink JR Peters B Southwood S Cerundolo V Grey H Yewdell JW Sette A HLA-A*0201, HLA-A*1101, and HLA-B*0702 transgenic mice recognize numerous poxvirus determinants from a wide variety of viral gene products. Journal of Immunology (Baltimore, Md.: 1950) 2005 175 5504 5515 16210659 10.4049/jimmunol.175.8.5504

[B66] Bhadra R Sandhya S Abhinandan KR Chakrabarti S Sowdhamini R Srinivasan N Cascade PSI-BLAST web server: a remote homology search tool for relating protein domains. Nucleic Acids Research 2006 34 W143 146 10.1093/nar/gkl157 16844978 PMC1538780

[B67] Melvin I Weston J Leslie C Noble WS RANKPROP: a web server for protein remote homology detection. Bioinformatics (Oxford, England) 2009 25 121 122 10.1093/bioinformatics/btn567 18990723 PMC2638939

[B68] Biegert A Söding J Sequence context-specific profiles for homology searching. Proceedings of the National Academy of Sciences of the United States of America 2009 106 3770 3775 10.1073/pnas.0810767106 19234132 PMC2645910

[B69] Querec TD Akondy RS Lee EK Cao W Nakaya HI Teuwen D Pirani A Gernert K Deng J Marzolf B Kennedy K Wu H Bennouna S Oluoch H Miller J Vencio RZ Mulligan M Aderem A Ahmed R Pulendran B Systems biology approach predicts immunogenicity of the yellow fever vaccine in humans. Nature Immunology 2009 10 116 125 10.1038/ni.1688 19029902 PMC4049462

[B70] Ressing ME Horst D Griffin BD Tellam J Zuo J Khanna R Rowe M Wiertz EJHJ Epstein-Barr virus evasion of CD8(+) and CD4(+) T cell immunity via concerted actions of multiple gene products. Seminars in Cancer Biology 2008 18 397 408 10.1016/j.semcancer.2008.10.008 18977445

[B71] Caldwell RG Wilson JB Anderson SJ Longnecker R Epstein-Barr virus LMP2A drives B cell development and survival in the absence of normal B cell receptor signals. Immunity 1998 9 405 411 10.1016/S1074-7613(00)80623-8 9768760

[B72] Miller CL Burkhardt AL Lee JH Stealey B Longnecker R Bolen JB Kieff E Integral membrane protein 2 of Epstein-Barr virus regulates reactivation from latency through dominant negative effects on protein-tyrosine kinases. Immunity 1995 2 155 166 10.1016/S1074-7613(95)80040-9 7895172

[B73] Graham JP Moore CR Bishop GA Roles of the TRAF2/3 binding site in differential B cell signaling by CD40 and its viral oncogenic mimic, LMP1. Journal of Immunology (Baltimore, Md.: 1950) 2009 183 2966 2973 19667091 10.4049/jimmunol.0900442 PMC2747101

[B74] Lee DY Sugden B The latent membrane protein 1 oncogene modifies B-cell physiology by regulating autophagy. Oncogene 2008 27 2833 2842 10.1038/sj.onc.1210946 18037963

[B75] Halary F Amara A Lortat-Jacob H Messerle M Delaunay T Houlès C Fieschi F Arenzana-Seisdedos F Moreau JF Déchanet-Merville J Human cytomegalovirus binding to DC-SIGN is required for dendritic cell infection and target cell trans-infection. Immunity 2002 17 653 664 10.1016/S1074-7613(02)00447-8 12433371

[B76] Naniche D Varior-Krishnan G Cervoni F Wild TF Rossi B Rabourdin-Combe C Gerlier D Human membrane cofactor protein (CD46) acts as a cellular receptor for measles virus. Journal of Virology 1993 67 6025 6032 8371352 10.1128/jvi.67.10.6025-6032.1993 PMC238023

[B77] Santoro F Kennedy PE Locatelli G Malnati MS Berger EA Lusso P CD46 is a cellular receptor for human herpesvirus 6. Cell 1999 99 817 827 10.1016/S0092-8674(00)81678-5 10619434

[B78] Kemper C Chan AC Green JM Brett KA Murphy KM Atkinson JP Activation of human CD4+ cells with CD3 and CD46 induces a T-regulatory cell 1 phenotype. Nature 2003 421 388 392 10.1038/nature01315 12540904

[B79] He Y Mou Z Li W Liu B Fu T Zhao S Xiang D Wu Y Identification of IMPDH2 as a tumor-associated antigen in colorectal cancer using immunoproteomics analysis. International Journal of Colorectal Disease 2009 24 1271 1279 10.1007/s00384-009-0759-2 19597826

[B80] Tobiume K Saitoh M Ichijo H Activation of apoptosis signal-regulating kinase 1 by the stress-induced activating phosphorylation of pre-formed oligomer. Journal of Cellular Physiology 2002 191 95 104 10.1002/jcp.10080 11920685

[B81] Chen Y McPhie DL Hirschberg J Neve RL The amyloid precursor protein-binding protein APP-BP1 drives the cell cycle through the S-M checkpoint and causes apoptosis in neurons. The Journal of Biological Chemistry 2000 275 8929 8935 10.1074/jbc.275.12.8929 10722740

[B82] Subramanian RP Geraghty RJ Herpes simplex virus type 1 mediates fusion through a hemifusion intermediate by sequential activity of glycoproteins D, H, L, and B. Proceedings of the National Academy of Sciences of the United States of America 2007 104 2903 2908 10.1073/pnas.0608374104 17299053 PMC1815279

[B83] Reimer JJ Backovic M Deshpande CG Jardetzky T Longnecker R Analysis of Epstein-Barr virus glycoprotein B functional domains via linker insertion mutagenesis. Journal of Virology 2009 83 734 747 10.1128/JVI.01817-08 18987135 PMC2612382

[B84] Backovic M Leser GP Lamb RA Longnecker R Jardetzky TS Characterization of EBV gB indicates properties of both class I and class II viral fusion proteins. Virology 2007 368 102 113 10.1016/j.virol.2007.06.031 17655906 PMC2131761

[B85] Neuhierl B Feederle R Hammerschmidt W Delecluse HJ Glycoprotein gp110 of Epstein-Barr virus determines viral tropism and efficiency of infection. Proceedings of the National Academy of Sciences of the United States of America 2002 99 15036 15041 10.1073/pnas.232381299 12409611 PMC137540

[B86] Loret S Guay G Lippé R Comprehensive characterization of extracellular herpes simplex virus type 1 virions. Journal of Virology 2008 82 8605 8618 10.1128/JVI.00904-08 18596102 PMC2519676

[B87] Padula ME Sydnor ML Wilson DW Isolation and preliminary characterization of herpes simplex virus 1 primary enveloped virions from the perinuclear space. Journal of Virology 2009 83 4757 4765 10.1128/JVI.01927-08 19279117 PMC2682069

[B88] Sollner J Grohmann R Rapberger R Perco P Lukas A Mayer B Analysis and prediction of protective continuous B-cell epitopes on pathogen proteins. Immunome Research 2008 4 1 10.1186/1745-7580-4-1 18179690 PMC2244602

[B89] Dosztányi Z Mészáros B Simon I ANCHOR: web server for predicting protein binding regions in disordered proteins. Bioinformatics 2009 25 20 2745 2746 10.1093/bioinformatics/btp518 19717576 PMC2759549

[B90] Panchenko AR Kondrashov F Bryant S Prediction of functional sites by analysis of sequence and structure conservation. Protein Science: A Publication of the Protein Society 2004 13 884 892 15010543 10.1110/ps.03465504 PMC2280064

[B91] Schwarz R Seibel PN Rahmann S Schoen C Huenerberg M Müller-Reible C Dandekar T Karchin R Schultz J Müller T Detecting species-site dependencies in large multiple sequence alignments. Nucleic Acids Research 2009 37 5959 5968 10.1093/nar/gkp634 19661281 PMC2764451

[B92] Sankararaman S Kolaczkowski B Sjölander K INTREPID: a web server for prediction of functionally important residues by evolutionary analysis. Nucleic Acids Research 2009 37 W390 395 10.1093/nar/gkp339 19443452 PMC2703888

[B93] Marchler-Bauer A Anderson JB Cherukuri PF DeWeese-Scott C Geer LY Gwadz M He S Hurwitz DI Jackson JD Ke Z Lanczycki CJ Liebert CA Liu C Lu F Marchler GH Mullokandov M Shoemaker BA Simonyan V Song JS Thiessen PA Yamashita RA Yin JJ Zhang D Bryant SH CDD: a Conserved Domain Database for protein classification. Nucleic Acids Research 2005 33 D192 196 10.1093/nar/gki069 15608175 PMC540023

[B94] Rawlings ND Barrett AJ Bateman A MEROPS: the peptidase database. Nucleic Acids Research 2009 9847218 10.1093/nar/27.1.325 PMC148173

[B95] Toussaint NC Dönnes P Kohlbacher O A mathematical framework for the selection of an optimal set of peptides for epitope-based vaccines. PLoS Computational Biology 2008 4 e1000246 10.1371/journal.pcbi.1000246 19112482 PMC2588662

[B96] Basha G Lizée G Reinicke AT Seipp RP Omilusik KD Jefferies WA MHC class I endosomal and lysosomal trafficking coincides with exogenous antigen loading in dendritic cells. PloS One 2008 3 e3247 10.1371/journal.pone.0003247 18802471 PMC2532750

[B97] Kotturi MF Assarsson E Peters B Grey H Oseroff C Pasquetto V Sette A Of mice and humans: how good are HLA transgenic mice as a model of human immune responses? Immunome Research 2009 5 3 10.1186/1745-7580-5-3 19534819 PMC2702351

[B98] Assarsson E Sidney J Oseroff C Pasquetto V Bui H Frahm N Brander C Peters B Grey H Sette A A quantitative analysis of the variables affecting the repertoire of T cell specificities recognized after vaccinia virus infection. Journal of Immunology (Baltimore, Md.: 1950) 2007 178 7890 7901 17548627 10.4049/jimmunol.178.12.7890

[B99] Overstreet MG Freyberger H Cockburn IA Chen YC Tse SW Zavala F CpG-enhanced CD8+ T cell responses to peptide immunization are severely inhibited by B cells. Eur J Immunol 2010 40 124 133 10.1002/eji.200939493 19830730 PMC2848446

[B100] Ishizuka J Grebe K Shenderov E Peters B Chen Q Peng Y Wang L Dong T Pasquetto V Oseroff C Sidney J Hickman H Cerundolo V Sette A Bennink JR McMichael A Yewdell JW Quantitating T cell cross-reactivity for unrelated peptide antigens. Journal of Immunology (Baltimore, Md.: 1950) 2009 183 4337 4345 19734234 10.4049/jimmunol.0901607 PMC2762195

[B101] Verma D Swaminathan S Epstein-Barr virus SM protein functions as an alternative splicing factor. Journal of Virology 2008 82 7180 7188 10.1128/JVI.00344-08 18463151 PMC2446978

[B102] Seib KL Dougan G Rappuoli R The key role of genomics in modern vaccine and drug design for emerging infectious diseases. PLoS Genetics 2009 5 e1000612 10.1371/journal.pgen.1000612 19855822 PMC2752168

[B103] Peters B Sidney J Bourne P Bui H Buus S Doh G Fleri W Kronenberg M Kubo R Lund O Nemazee D Ponomarenko JV Sathiamurthy M Schoenberger S Stewart S Surko P Way S Wilson S Sette A The immune epitope database and analysis resource: from vision to blueprint. PLoS Biology 2005 3 e91 10.1371/journal.pbio.0030091 15760272 PMC1065705

[B104] de Jesus O Smith PR Spender LC Elgueta Karstegl C Niller HH Huang D Farrell PJ Updated Epstein-Barr virus (EBV) DNA sequence and analysis of a promoter for the BART (CST, BARF0) RNAs of EBV. The Journal of General Virology 2003 84 1443 1450 10.1099/vir.0.19054-0 12771413

[B105] Dolan A Addison C Gatherer D Davison AJ McGeoch DJ The genome of Epstein-Barr virus type 2 strain AG876. Virology 2006 350 164 170 10.1016/j.virol.2006.01.015 16490228

[B106] Eswar N Webb B Marti-Renom MA Madhusudhan MS Eramian D Shen M Pieper U Sali A Comparative protein structure modeling using Modeller. Current protocols in bioinformatics / editoral board, Andreas D. Baxevanis ... [et al.] 2006 Chapter 5:Unit 5.6 18428767 10.1002/0471250953.bi0506s15 PMC4186674

[B107] Berman H Henrick K Nakamura H Announcing the worldwide Protein Data Bank. Nature Structural Biology 2003 10 980 10.1038/nsb1203-980 14634627

[B108] Weichenberger CX Sippl MJ NQ-Flipper: validation and correction of asparagine/glutamine amide rotamers in protein crystal structures. Bioinformatics (Oxford, England) 2006 22 1397 1398 10.1093/bioinformatics/btl128 16595557

[B109] DeLano W The PyMOL User's Manual. DeLano Scientific 2002 Palo Alto, CA, USA

[B110] Mészáros B Simon I Dosztányi Z Prediction of protein binding regions in disordered proteins. PLoS Computational Biology 2009 5 e1000376 10.1371/journal.pcbi.1000376 19412530 PMC2671142

[B111] Sweredoski MJ Baldi P COBEpro: a novel system for predicting continuous B-cell epitopes. Protein Engineering, Design & Selection: PEDS 2009 22 113 120 10.1093/protein/gzn075 19074155 PMC2644406

[B112] El-Manzalawy Y Dobbs D Honavar V Predicting flexible length linear B-cell epitopes. Computational Systems Bioinformatics / Life Sciences Society. Computational Systems Bioinformatics Conference 2008 7 121 132 19642274 PMC3400678

[B113] Haste Andersen P Nielsen M Lund O Prediction of residues in discontinuous B-cell epitopes using protein 3D structures. Protein Science: A Publication of the Protein Society 2006 15 2558 2567 17001032 10.1110/ps.062405906 PMC2242418

[B114] Ponomarenko J Bui H Li W Fusseder N Bourne PE Sette A Peters B ElliPro: a new structure-based tool for the prediction of antibody epitopes. BMC Bioinformatics 2008 9 514 10.1186/1471-2105-9-514 19055730 PMC2607291

[B115] Jones DT Improving the accuracy of transmembrane protein topology prediction using evolutionary information. Bioinformatics (Oxford, England) 2007 23 538 544 10.1093/bioinformatics/btl677 17237066

[B116] Krogh A Larsson B von Heijne G Sonnhammer EL Predicting transmembrane protein topology with a hidden Markov model: application to complete genomes. Journal of Molecular Biology 2001 305 567 580 10.1006/jmbi.2000.4315 11152613

[B117] Sonnhammer EL von Heijne G Krogh A A hidden Markov model for predicting transmembrane helices in protein sequences. Proceedings / ... International Conference on Intelligent Systems for Molecular Biology ; ISMB. International Conference on Intelligent Systems for Molecular Biology 1998 6 175 182 9783223

[B118] Adamczak R Porollo A Meller J Accurate prediction of solvent accessibility using neural networks-based regression. Proteins 2004 56 753 767 10.1002/prot.20176 15281128

[B119] Adamczak R Porollo A Meller J Combining prediction of secondary structure and solvent accessibility in proteins. Proteins 2005 59 467 475 10.1002/prot.20441 15768403

[B120] Julenius K Mølgaard A Gupta R Brunak S Prediction, conservation analysis, and structural characterization of mammalian mucin-type O-glycosylation sites. Glycobiology 2005 15 153 164 10.1093/glycob/cwh151 15385431

[B121] Prediction of N-glycosylation sites in human proteins. http://www.cbs.dtu.dk/services/NetNGlyc/

[B122] Matthews BW Comparison of the predicted and observed secondary structure of T4 phage lysozyme. Biochimica Et Biophysica Acta 1975 405 442 451 1180967 10.1016/0005-2795(75)90109-9

[B123] Baldi P Brunak S Chauvin Y Andersen CA Nielsen H Assessing the accuracy of prediction algorithms for classification: an overview. Bioinformatics (Oxford, England) 2000 16 412 424 10.1093/bioinformatics/16.5.412 10871264

[B124] Buus S Lauemøller SL Worning P Kesmir C Frimurer T Corbet S Fomsgaard A Hilden J Holm A Brunak S Sensitive quantitative predictions of peptide-MHC binding by a 'Query by Committee' artificial neural network approach. Tissue Antigens 2003 62 378 384 10.1034/j.1399-0039.2003.00112.x 14617044

[B125] Nielsen M Lundegaard C Worning P Lauemøller SL Lamberth K Buus S Brunak S Lund O Reliable prediction of T-cell epitopes using neural networks with novel sequence representations. Protein Science: A Publication of the Protein Society 2003 12 1007 1017 12717023 10.1110/ps.0239403 PMC2323871

[B126] Larsen MV Lundegaard C Lamberth K Buus S Lund O Nielsen M Large-scale validation of methods for cytotoxic T-lymphocyte epitope prediction. BMC Bioinformatics 2007 8 424 10.1186/1471-2105-8-424 17973982 PMC2194739

[B127] Nielsen M Lundegaard C Lund O Prediction of MHC class II binding affinity using SMM-align, a novel stabilization matrix alignment method. BMC Bioinformatics 2007 8 238 10.1186/1471-2105-8-238 17608956 PMC1939856

[B128] Nielsen M Lundegaard C Lund O Keşmir C The role of the proteasome in generating cytotoxic T-cell epitopes: insights obtained from improved predictions of proteasomal cleavage. Immunogenetics 2005 57 33 41 10.1007/s00251-005-0781-7 15744535

[B129] Hoof I Peters B Sidney J Pedersen LE Sette A Lund O Buus S Nielsen M NetMHCpan, a method for MHC class I binding prediction beyond humans. Immunogenetics 2009 61 1 13 10.1007/s00251-008-0341-z 19002680 PMC3319061

[B130] Nielsen M Lundegaard C Blicher T Peters B Sette A Justesen S Buus S Lund O Quantitative predictions of peptide binding to any HLA-DR molecule of known sequence: NetMHCIIpan. PLoS Computational Biology 2008 4 e1000107 10.1371/journal.pcbi.1000107 18604266 PMC2430535

[B131] Senger M Rice P Bleasby A Uludag M Soaplab: open source web services framework for Bioinformatics programs. In The 10th Annual Bioinformatics Open Source Conference 2009 168 27 28

[B132] Holland RCG Down TA Pocock M Prlić A Huen D James K Foisy S Dräger A Yates A Heuer M Schreiber MJ BioJava. Bioinformatics 2008 24 2096 2097 10.1093/bioinformatics/btn397 18689808 PMC2530884

[B133] Drummond A Strimmer K PAL: an object-oriented programming library for molecular evolution and phylogenetics. Bioinformatics 2001 17 662 663 10.1093/bioinformatics/17.7.662 11448888

[B134] Edgar RC MUSCLE: multiple sequence alignment with high accuracy and high throughput. Nucleic Acids Research 2004 32 1792 1797 10.1093/nar/gkh340 15034147 PMC390337

[B135] Jones DT Protein secondary structure prediction based on position-specific scoring matrices. Journal of Molecular Biology 1999 292 195 202 10.1006/jmbi.1999.3091 10493868

